# Proinflammatory Cytokines Perturb Mouse and Human Pancreatic Islet Circadian Rhythmicity and Induce Uncoordinated β-Cell Clock Gene Expression via Nitric Oxide, Lysine Deacetylases, and Immunoproteasomal Activity

**DOI:** 10.3390/ijms22010083

**Published:** 2020-12-23

**Authors:** Phillip Alexander Keller Andersen, Volodymyr Petrenko, Peter Horskjær Rose, Melissa Koomen, Nico Fischer, Seyed Mojtaba Ghiasi, Tina Dahlby, Charna Dibner, Thomas Mandrup-Poulsen

**Affiliations:** 1 Department of Biomedical Sciences, Faculty of Health and Medical Sciences, University of Copenhagen, 3 Blegdamsvej, DK-2200 Copenhagen N, Denmark; phillip.andersen@sund.ku.dk (P.A.K.A.); horskjaerrose@gmail.com (P.H.R.); melissa_koomen@hotmail.com (M.K.); nico.fischer98@hotmail.com (N.F.); s.ghiasi@imperial.ac.uk (S.M.G.); tina.dahlby@hest.ethz.ch (T.D.); 2 Division of Endocrinology, Diabetes, Nutrition and Patient Education, Department of Cell Physiology and Metabolism, Diabetes Center, Faculty of Medicine, University of Geneva, D05.2147c Rue Michel-Servet, 1 CH-1211 Geneva 4, Switzerland; volodymyr.petrenko@unige.ch (V.P.); charna.dibner@hcuge.ch (C.D.)

**Keywords:** chronobiology, diabetes, epigenetics, immuno-metabolism, nitric oxide synthase

## Abstract

Pancreatic β-cell-specific clock knockout mice develop β-cell oxidative-stress and failure, as well as glucose-intolerance. How inflammatory stress affects the cellular clock is under-investigated. Real-time recording of Per2:luciferase reporter activity in murine and human pancreatic islets demonstrated that the proinflammatory cytokine interleukin-1β (IL-1β) lengthened the circadian period. qPCR-profiling of core clock gene expression in insulin-producing cells suggested that the combination of the proinflammatory cytokines IL-1β and interferon-γ (IFN-γ) caused pronounced but uncoordinated increases in mRNA levels of multiple core clock genes, in particular of reverse-erythroblastosis virus α *(Rev-erbα)*, in a dose- and time-dependent manner. The REV-ERBα/β agonist SR9009, used to mimic cytokine-mediated *Rev-erbα* induction, reduced constitutive and cytokine-induced brain and muscle arnt-like 1 (*Bmal1*) mRNA levels in INS-1 cells as expected. SR9009 induced reactive oxygen species (ROS), reduced insulin-1/2 (*Ins-1/2*) mRNA and accumulated- and glucose-stimulated insulin secretion, reduced cell viability, and increased apoptosis levels, reminiscent of cytokine toxicity. In contrast, low (<5,0 μM) concentrations of SR9009 increased *Ins-1* mRNA and accumulated insulin-secretion without affecting INS-1 cell viability, mirroring low-concentration IL-1β mediated β-cell stimulation. Inhibiting nitric oxide (NO) synthesis, the lysine deacetylase HDAC3 and the immunoproteasome reduced cytokine-mediated increases in clock gene expression. In conclusion, the cytokine-combination perturbed the intrinsic clocks operative in mouse and human pancreatic islets and induced uncoordinated clock gene expression in INS-1 cells, the latter effect associated with NO, HDAC3, and immunoproteasome activity.

## 1. Introduction

All photosensitive organisms possess circadian clocks synchronizing the temporal organization of cell and system physiology with the rotation of the earth [1,2,3,4,5,6,7]. The master pacemaker of the mammalian circadian clock is the suprachiasmatic nucleus (SCN) of the hypothalamus, which is mainly timed by light input [8,9,10]. However, virtually all cells harbor self-sustained and cell-autonomous molecular clocks, which are normally driven by feeding rhythms and rest-activity cycles [11,12]. The peripheral clocks are synchronized by the dominant SCN clock and establish phase coherence in the body through molecular pathways that are coordinated by transcriptional feed-forward and feed-back loops regulating oscillatory expression of core clock genes [8,12,13,14].

Circadian oscillation is essential in priming the function of several metabolic processes in anticipation of diurnal cycling [7,12,15,16,17]. This is illustrated by the fact that perturbation of the circadian clock causes insulin resistance and β-cell secretory dysfunction [1,15,18,19,20,21,22] and that mice with transgenic global or pancreatic β-cell specific clock disruption display glucose intolerance and β-cell apoptosis due to oxidative stress [15,23]. It has recently emerged that immune function, inflammation, metabolism, and tissue remodeling are closely interconnected with the circadian system, and that the connection between these physiological systems occurs at multiple levels and in a bi-directional fashion [20,24,25,26,27,28,29,30]. Accordingly, proinflammatory nuclear factor of kappa light polypeptide gene enhancer in B-cells (NF-κB) signaling induces the expression of several core clock genes by inhibiting clock repressors, including the period (*Per*), cryptochrome (*Cry*), and reverse-erythroblastosis virus (*Rev-erb*) genes, and genome-wide recruitment of the circadian locomotor output cycles kaput (CLOCK)/ brain and muscle arnt-like 1 (BMAL1) transcriptional complex co-activates NF-κB, thereby contributing to transcriptional re-programming in response to inflammatory stimuli [31].

Pancreatic islet inflammation has been implicated in the pathogenesis of Type 1 diabetes [32,33,34,35]. Exposure of non-synchronized pancreatic β-cells to proinflammatory cytokines triggers mitogen-activated protein kinases (MAPK) and NF-κB signaling leading to oxidative stress and activation of the intrinsic (mitochondrial) apoptotic pathway [36]. Circadian misalignment adversely affects glycemic control in Type 1 diabetic subjects by unclear mechanisms [37,38]. One possibility is that clock disruption adversely affects residual functional β-cell mass found in most adult patients with even long-standing Type 1 diabetes by impeding on the balance between β-cell regeneration and inflammatory destruction, as residual β-cell function contributes significantly to glycemic stability [39].

Yet, how inflammatory stress affects the β-cell circadian clock is an under-investigated area of research, and so far, in vitro studies of cytokine effects on β-cell fate have all been conducted on non-synchronized models. Only very recently [40] it was reported that out of the proinflammatory cytokines interleukin-1β (IL-1β), tumor necrosis factor α (TNFα), interleukin 6 (IL-6), and interferon-γ (IFN-γ) tested individually, IL-1β dampened circadian amplitude and accelerated β-cell circadian rhythm recorded in vitro by time-lapse bioluminescence assay in non-synchronized islets, and reduced *Bmal1* expression in a sirtuin-1 dependent manner in INS-1 832/13 cells. IL-1β also inhibited retinoic acid receptor-related orphan receptor α (RORα) protein expression, associated with induction of REV-ERBα, whereas TNFα or IL-6 reduced *Bmal1*, and IL-6 or IFN-γ reduced *Clock* gene expression.

The present study aimed at clarifying this important knowledge gap, with the overall hypothesis that the impact of inflammatory cytokines on circadian clock function depends on the cytokine context, i.e., the concentration, duration of exposure, and combination of cytokines. Further, we wished to explore common mechanistic pathways in the regulation of inflammatory signaling and of clock biology, in particular the role of nitroxidative stress, the lysine deacetylase HDAC3, and the cytokine-inducible immunoproteasome, all known to be either negatively or positively involved in cytokine-signaling in β-cells [35,36,41]. We demonstrate here that proinflammatory cytokines perturbed the intrinsic β-cell clock in murine and human islets expressing Per2-luciferase reporter and induced uncoordinated clock gene expression in INS-1 cells, the latter effect associated with nitric oxide (NO), histone deacetylase 3 (HDAC3), and immunoproteasome activity.

## 2. Results

### 2.1. IL-1β Affects Circadian Clockwork in Murine and Human Pancreatic Islets

**Figure 1:**
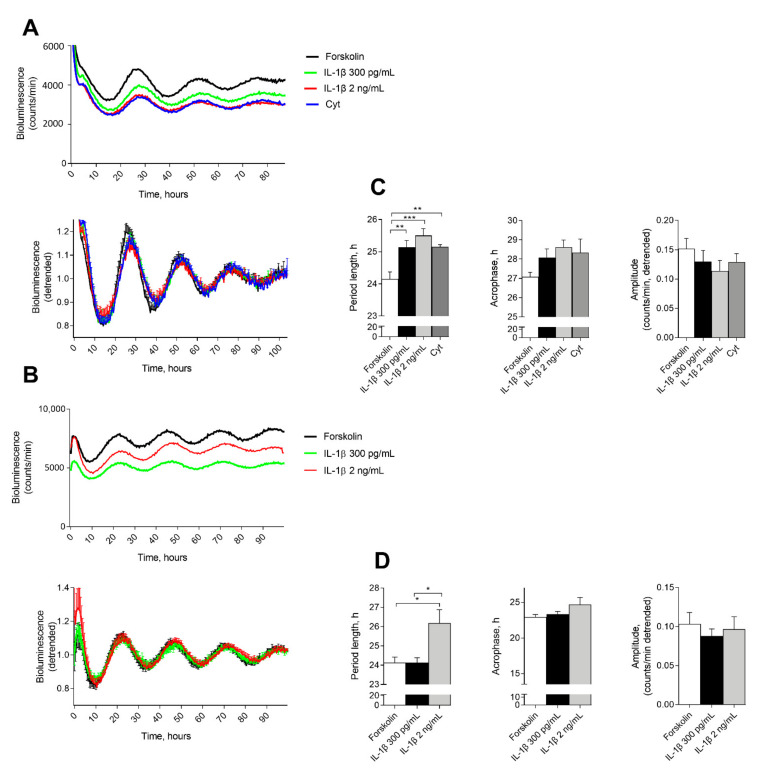
Proinflammatory cytokines alter the molecular clockwork in synchronized mouse and human Per2-luciferase (Per2-luc) reporter islets. (**A**,**B**) Average raw (top panels) and detrended (bottom panels) Per2-luc oscillatory profiles recorded from forskolin-synchronized mouse (**A**, *n* = 5 of independent islet isolations with 2–3 animals per isolation) and human (**B**, *n* = 4 of human donors) islets in the presence of interleukin-1β (IL-1β) in the indicated concentrations or a combination of 300 pg/mL IL-1β and 0.2 ng/mL interferon-γ (IFN- γ) (Cyt). (**C**,**D**) Average period length, acrophase, and amplitude were calculated based on the detrended bioluminescence values for mouse (**C**) and human (**D**) islets. Values are means or means ± SEM. Statistics are one-way ANOVA with Tukey corrected multiple comparisons. Significance levels were annotated as follows: * = *p*-value < 0.05, ** = *p*-value < 0.01, *** = *p*-value < 0.001.

To assess the effect of proinflammatory cytokines on the molecular clocks operative in mouse and human islets, we recorded bioluminescence profiles of Per2-luciferase (Per2-luc) from reporter mouse islets (Figure 1A), and from human islets transduced with Per2-luc lentivectors (Figure 1B). Isolated mouse or human islets were attached to the dish, synchronized with a forskolin pulse, and bioluminescence was subsequently recorded in the presence of vehicle, 300 pg/mL or 2 ng/mL of IL-1β during several consecutive days. In mouse islets, IL-1β induced a dose-dependent reduction in overall expression levels of Per2-luc reporter. IFN-γ exhibited no additional effect when combined with IL-1β (Figure 1A). Analysis of rhythmic parameters based on the detrended values demonstrated significant and dose-dependent lengthening of the reporter circadian period that stemmed from the application of low and high concentrations of IL-1β (1 h and 1.5 h, respectively) (Figure 1C). A trend for delay was observed for acrophase, whereas amplitude seemed slightly decreased in the mouse islet treated with IL-1β, although these trends did not reach statistical significance (Figure 1C). In human islets, whereas low concentration of IL-1β resulted in no significant effect on the islet circadian characteristics, application of IL-1β at high concentration led to significant lengthening of circadian period (Figure 1B,D). Surprisingly, low but not high concentration of IL-1β caused a reduction in overall Per2-luc expression levels (Figure 1D).

Taken together, these data indicate that IL-1β significantly lengthens the circadian period of the oscillatory profiles of the islet clock in synchronized mouse islets in a dose-dependent manner. The human islet clocks only exhibited similar period alterations in the presence of high levels of IL-1β, as also observed for most other IL-1β-induced effects in human islets [36]. No significant effect of cytokine exposure on viability was observed under these conditions either in human or mouse islets (Figure S1), suggesting that the effects of IL-1β on the circadian clock in synchronized pancreatic islet cells are not attributed to the cytotoxicity of this inflammatory cytokine.

### 2.2. Proinflammatory Cytokines Induce Marked but Uncoordinated Expression of Core Clock Genes in Insulin Producing Cells

**Figure 2:**
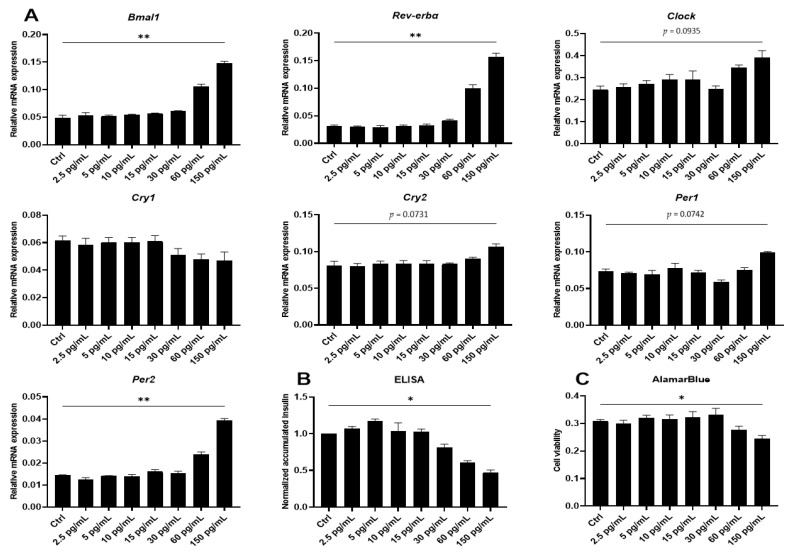
IL-1β differentially affects clock gene expression in non-synchronized INS-1 cells in a dose-dependent manner, associated with loss of cell function and viability**.** INS-1 cells were exposed to increasing concentrations of mouse IL-1β for 12 h. Effects on clock gene expression were evaluated (**A**), while function and cell viability were assessed through quantification of accumulated insulin (ELISA) (**B**) and AlamarBlue (**C**), respectively. Relative mRNA expression is calculated using hypoxanthine-guanine phosphoribosyltransferase (*Hprt1*) and *β-Actin* as reference genes. Values are means ± SEM (*n* = 3). Statistics are repeated measures one-way ANOVA with the *p*-values represented by symbols above the line. Significance levels were annotated as follows: * = *p*-value < 0.05, ** = *p*-value < 0.01.

The experiments on synchronized murine and human reporter islets were limited to the study of only two concentrations of IL-1β but did indicate that this cytokine exerts its effects on the islet circadian period length in an apparent dose-dependent manner (Figure 1C). IL-1β is known to have a biphasic mode of action, where low concentrations stimulate β-cell function and high concentrations reduce viability and function [36]. To determine more carefully the dose-dependency of IL-1β action on the intrinsic clock, the expression of key clock genes in non-synchronized INS-1 cells after IL-1β treatment for 12 h was evaluated. IL-1β treatment robustly induced the expression of *Bmal1*, *Rev-erbα*, and *Per2*, with a trend for induction of *Clock*, *Per1*, and *Cry2*, while *Cry1* expression was not significantly affected (Figure 2A). The increase in clock gene expression mirrored the cytotoxic effects associated with concentrations of IL-1β above 30 pg/mL, evidenced by a reduction in accumulated insulin release (Figure 2B) and cell viability (Figure 2C) at these concentrations after 12 h. However, low concentrations (2.5 and 5 pg/mL) associated with the stimulatory effect of IL-1β (Figure 2B) did not affect the expression of clock genes.

**Figure 3:**
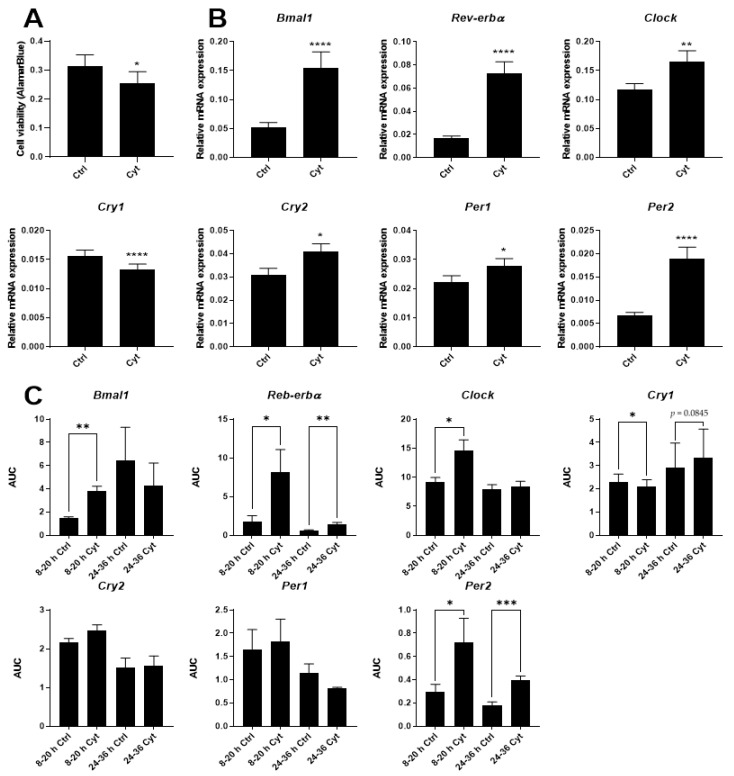
IL-1β and IFN-γ mediate differential but uncoordinated alterations in the expression of core clock genes in non-synchronized INS-1 cells. INS-1 cells were exposed to 150 pg/mL mouse IL-1β + 0.1 ng/mL rat IFN-γ (Cyt) for either 12 h (**A**,**B**) or at 4 h intervals from 8–36 h (**C**). (**A**) Cell viability was assessed using AlamarBlue (*n* = 4). (B) Relative mRNA expression is calculated using 5S rRNA and *Hprt1* as reference genes. Values are means ± SEM (*n* = 6). (**C**) Relative mRNA expression is calculated using *Hprt1* as reference genes. The time-dependent response of cytokine treatment was divided into two intervals consisting of the 8–20-h and 24–36-h intervals, from which areas under the curve (AUC) were calculated and used for subsequent statistical analysis. Values are means ± SEM (*n* = 5). Statistics are paired Student’s *t*-test. Significance levels were annotated as follows: * = *p*-value < 0.05, ** = *p*-value < 0.01, *** = *p*-value < 0.001, **** = *p*-value < 0.0001.

Having demonstrated that IL-1β by itself dose-dependently induces expression of several clock genes, associated with a decrease in β-cell viability and function, the effects of the combination of IL-1β and IFN-γ was investigated. Employing a combination of cytokine concentrations yielded a decrease in cell viability after 12 h (Figure 3A) similar to that obtained by 150 pg/mL IL-1β alone, as well as induction of *Bmal1*, *Rev-erbα*, *Clock*, *Cry2*, *Per1*, and *Per2*, and a decrease in *Cry1* (Figure 3B). However, the combination of IL-1β and IFN-γ did not have a synergistic effect on clock gene expression in INS-1 cells (compare Figure 2A and Figure 3B). Yet, because the combination of these two cytokines provides a more faithful model for islet inflammation in Type 1 diabetes, where both innate and adaptive cytokine networks are involved, and since activation of both the IL-1 and IFN-γ receptor yields the relevant signaling background for the action of the employed inhibitors, affecting pathways where synergy or additive effects have been described, the combination of the two cytokines was used in the mechanistic experiments.

We next examined the time-dependency of the clock gene expressions by sampling RNA with four-hour-intervals between 8 and 36 h of exposure to IL-1β and IFN-γ (Figure S2A). *Bmal1*, *Rev-erbα*, *Clock*, *Per2*, and *Cry1* were markedly and differentially affected by cytokines in the 8–20 h-interval, while only *Rev-erbα* and *Per2* were significantly affected in the 24–36 h-interval (Figure S2A and Figure 3C).

The early induction of several clock genes (Figure S2A) also observed using other synchronizing signals [42,43] might indicate that cytokine exposure exerts a Zeitgeber effect in non-synchronized cells. However, cytokine exposure for two hours did not increase *Per1* and *Per2* expression (*p* = 0.61 and 0.3304 respectively) (Figure S2B). In contrast, a one-hour forskolin pulse did induce *Per1* (and trended to induce *Per2*) expression, indicating that the cytokine combination does not provide a “canonical” entrainment signal. Moreover, cytokines did not interfere with a forskolin synchronization signal, when the forskolin pulse was administrated to cells either pre-exposed (Figure S2C) to or co-incubated (Figure S2B) with cytokines. As positive controls for cytokine signaling increases in early (*Fas* and Nuclear factor of kappa light polypeptide gene enhancer in B-cells inhibitor α (*IκBα*)) and late (inducible nitric oxide synthase (*Inos*)) inflammatory response genes were demonstrated (Figure S2B,C). In summary, proinflammatory cytokines induce a broad but non-phasic expression of several central clock genes in a time- and dose-dependent manner in non-synchronized INS-1 cells, but do not appear to be a Zeitgeber or affect synchronization.

### 2.3. The REV-ERBα/β Agonist Inhibits Bmal1 in the Absence and Presence of Cytokines in Non-Synchronized Insulin-Producing Cells

**Figure 4:**
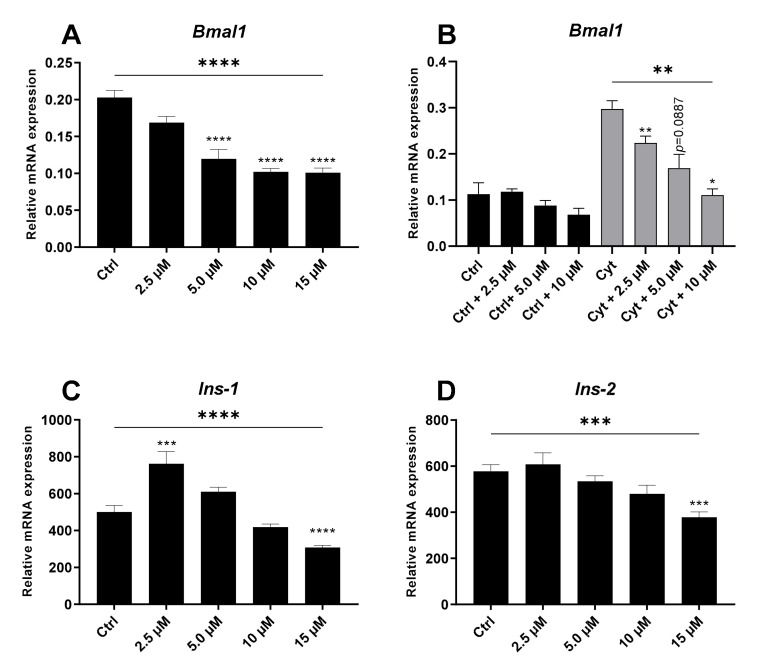
SR9009 dose- dependently decreases brain and muscle arnt-like 1 (*Bmal1*) and insulin 1/2 (*Ins-1/2*) expression in non-synchronized INS-1 cells. INS-1 cells were exposed to increasing doses of SR9009 alone for 24 h (**A**–**D**) or in combination with 150 pg/mL mouse IL-1β + 0.1 ng/mL rat IFN-γ (Cyt) for 12 h (**B**). Relative mRNA expression is calculated using *Hprt1* as reference gene. Values are mean ± SEM (*n* = 3–9). Statistics are one-way ANOVA with *p*-values represented by symbols above the line and with Dunnett’s corrected multiple comparisons to Ctrl (black bars) or to Cyt (gray bars). Significance levels were annotated as follows: * = *p*-value < 0.05, ** = *p*-value < 0.01, *** = *p*-value < 0.001, **** = *p*-value < 0.0001.

**Figure 5:**
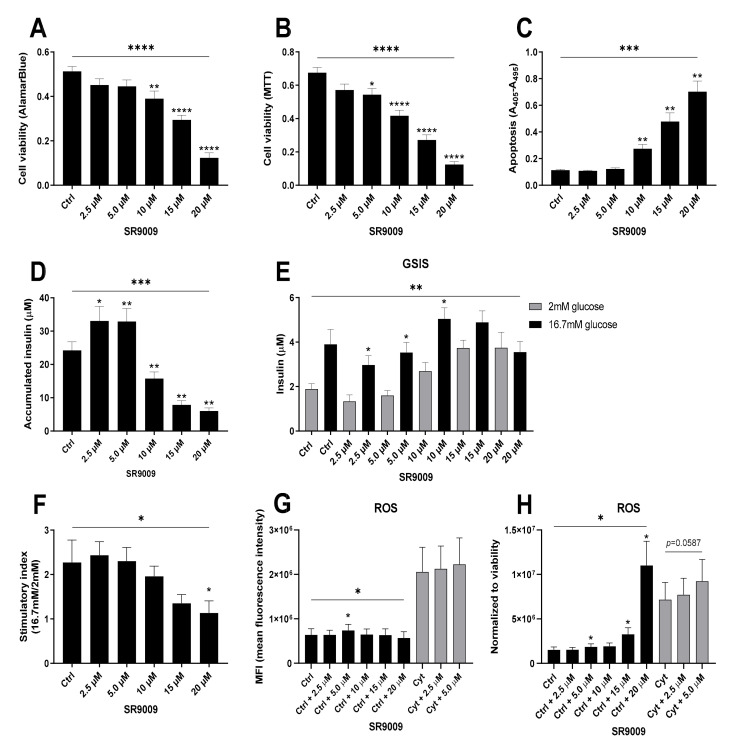
SR9009 reduces viability and function in a reactive oxygen species (ROS)-dependent manner in non-synchronized INS-1 cells. INS-1 cells were exposed to increasing concentrations of SR9009 alone or in combination with 150 pg/mL mouse IL-1β + 0.1 ng/mL rat IFN-γ (Cyt) for 24 h to assess the effect on cell viability (**A**,**B**), apoptosis levels (**C**), accumulated insulin (**D**), glucose stimulated insulin secretion (GSIS) (**E**,**F**) and ROS production (**G**,**H**). Values are mean ± SEM (*n* = 6–12). Statistics are one-way ANOVA with *p*-values represented by symbols above the line and with Dunnett’s corrected multiple comparisons to Ctrl (black bars) or to Cyt (gray bars), or with Bonferroni corrected multiple comparisons between similar concentrations of SR9009 (**E**), and post hoc paired Student’s *t*-test (**F**,**G**). Significance levels were annotated as follows: * = *p*-value < 0.05, ** = *p*-value < 0.01, *** = *p*-value < 0.001, **** = *p*-value < 0.0001.

Since proinflammatory cytokine-induced *Rev-erbα* expression was the most robust change in core clock gene expression (>5-fold) and associated with cytokine toxicity, we next wished to disentangle the effect of *Rev-erbα* induction from other cytokine-triggered signaling pathways to define the functional effects of isolated *Rev-erbα* induction. We took advantage of the small-molecule REV-ERBα/β agonist SR9009 and first verified that this agonist did indeed markedly and dose-dependently inhibit *Bmal1* in non-synchronized INS-1 cells as anticipated in the absence of inflammatory cytokines (Figure 4A). Of note, the agonist at 10 μM normalized cytokine-induced *Bmal1* expression (Figure 4B). We determined insulin gene expression (*Ins-1* and *Ins-2*) in the same samples used for *Bmal1* amplification. As shown in Figure 4C,D, 5 and 10 μM of SR9009 that markedly inhibited *Bmal1* expression did not significantly reduce *Ins-1/2* expression. Interestingly, 2.5 μM SR9009 that did not significantly reduce *Bmal1* expression stimulated *ins-1* expression, whereas only the highest SR9009 concentration inhibited expression of all three genes (Figure 4A–D). These observations indicate that the inhibitory action of REV-ERBα/β agonist on *Bmal1* was not due to a non-specific action on mRNA synthesis, and that modest *Bmal1* inhibition improved *ins-1* gene transcription.

### 2.4. The REV-ERBα/β Agonist Mimics Cytokine-Action in Non-Synchronized Insulin-Producing Cells

Since cytokines cause β-cell functional impairment and toxicity by triggering the mitochondrial (intrinsic) death pathway, we next investigated viability and mitochondrial function of INS-1 cells in the presence of SR9009. SR9009 dose-dependently impaired mitochondrial reducing capacity and cell viability as determined by both AlamarBlue and MTT assay (Figure 5A,B), but did not sensitize INS-1 cells to cytokine-induced mitochondrial impairment (Figure S3). Up to 10 μM of SR9009, inhibition of mitochondrial function was not accompanied by apoptosis, whereas at 10 μM and above, SR9009 increased apoptosis rates (Figure 5C). Interestingly, non-apoptotic concentrations of SR9009 increased accumulated insulin release (Figure 5D), reminiscent of the bimodal action of proinflammatory cytokines. Non-apoptotic concentrations of SR9009 did not affect glucose-stimulated insulin secretion (GSIS) (Figure 5E), whereas apoptotic concentrations tended to reduce the stimulatory index (SI) as expected (Figure 5F), caused by increases in both basal and glucose stimulated insulin secretion.

As SI only appeared to be reduced at high concentrations of SR9009, and no apoptosis was observed at 2.5–5 µM, the increase in accumulated insulin at low concentrations is not likely to be due to passive leakage, but could be due to glucose-independent regulation, e.g., increased levels of ROS [44]. At 5 µM SR9009 that increased accumulated insulin release, ROS levels were indeed increased (Figure 5G). When correcting for cell loss, ROS production by viable cells was robustly increased, and a trend for an additive effect on cytokine-mediated ROS production at low concentrations of SR9009 was observed (Figure 5H). These data suggest that modestly increased REV-ERBα activity, associated with decreased *Bmal1* expression, causes an increase in insulin transcription and secretion and ROS formation, while markedly increased REV-ERBα activity decreases mitochondrial function, viability, and insulin transcription and secretion in β-cells associated with a marked increase in ROS production.

### 2.5. Metabolic Stress Perturbs the Intrinsic β-Cell Clock but Differently From Inflammatory Stress

The observation that inflammatory stress can perturb the circadian clock in β-cells raised the question if this response is specific or shared with other β-cell stressors. Although both inflammatory and metabolic stress cause oxidative β-cell damage, glucolipotoxicity (GLT) does not utilize NF-κB as early immediate signal, but elicits the endoplasmic reticulum stress pathway and facilitates ROS production by enhancing iron import [45].

**Figure 6:**
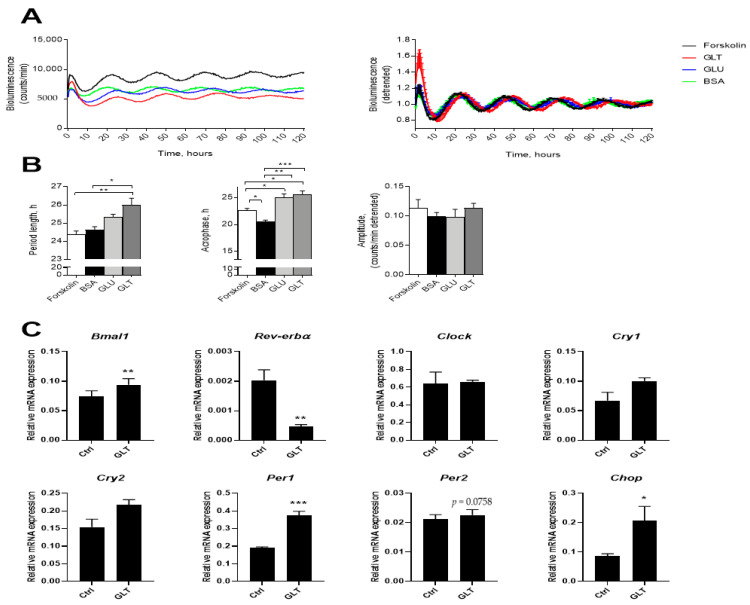
Glucolipotoxicity (GLT) alters clockwork in synchronized human Per2-luc reporter islets and clock gene expression in non-synchronized INS-1E cells. (**A**) Average raw (left panel) and detrended (right panel) Per2-luc oscillatory profiles recorded from forskolin-synchronized human islets (*n* = 3 human donors) exposed to a combination of 500 nM palmitate and 20 mmol glucose (GLT), to 20 mmol glucose (GLU), or to BSA (bovine serum albumin; palmitate carrier) alone. (**B**) Average period length, acrophase, and amplitude were calculated based on the detrended values. See also Figure S6. (**C**) INS-1E cells were exposed to 0.5 mM palmitate + 25 mM glucose (GLT) for 12 h. Relative mRNA expression was calculated using *Hprt1* as reference gene (*n* = 4). Values are means or means ± SEM. Statistics are one-way ANOVA with Tukey corrected multiple comparisons (**B**) or paired Student’s *t*-test (**C**). Significance levels were annotated as follows: * = *p*-value < 0.05, ** = *p*-value < 0.01, *** = *p*-value < 0.001.

To assess the impact of metabolic stressors on the molecular oscillators functional in human pancreatic islets, the islets were transduced with Per2-luc lentivectors, synchronized by forskolin, and treated either with 20 mmol glucose, or with a mix of glucose and palmitate to induce GLT. Bovine serum albumin (BSA) alone was added as the palmitate carrier control. All the substances slightly decreased the absolute levels of Per2-luc signal (Figure 6A). Quantification of rhythmic parameters based on the detrended values indicated significant lengthening of circadian period upon GLT treatment (Figure 6B left panel), similar to the effect of IL-1β (Figure 1D). While BSA application alone slightly advanced the circadian phase of Per2-luc oscillations, both GLT and high glucose delayed it (Figure 6B middle panel). No effect on circadian amplitude was recorded (Figure 6B, right panel). Of note, a trend for increased apoptosis in human islet cells was observed upon GLT condition, although it did not reach statistical significance (Figure S4A).

Concordantly, when we applied GLT treatment to the mouse islets bearing Per2-luc reporter and synchronized in vitro, a similar trend for phase delay and lengthening of the circadian period was observed (Figure S4B). In addition, the amplitude dampening paralleled with an enhanced apoptosis (Figure S4B,C) induced by GLT treatment in mouse islets as compared to the human counterparts.

In INS-1E cells, chosen for these experiments for their superior glucose-sensitivity relative to that of INS-1 cells, exposure to GLT markedly downregulated *Rev-erbα*, while upregulating *Bmal1* and *Per1* expression (a trend was observed for *Per2*) in non-synchronized INS-1E cells at 12 h (Figure 6C). These responses were associated with an increase in C/EBP homologous protein (*Chop*), confirming that GLT was effective in increasing ER stress (Figure 6C). In a time-response experiment probing gene expression at 6-h-intervals during a 24 period, the same tendency was observed (Figure S5). Thus, both inflammatory and metabolic stress perturb the intrinsic clock in β-cells, albeit via apparently different pathways.

### 2.6. Proinflammatory Cytokine-Mediated Expression of Clock Genes Depends on Lysine Deacetylase Activity, Nitric Oxide, and the Immunoproteasome in Insulin Producing Cells

Cytokine-induced β-cell damage and oxidative stress are mediated through MAPK and NF-κB signaling [35]. Lysine deacetylases (KDACs), and HDAC3 in particular, promote the harmful effect of NF-κB signaling by deacetylating p65 [46,47,48], thereby increasing its binding to inflammatory promotors such as iNOS and resulting in increased synthesis of NO and promotion of nitroxidative stress [49]. Notably, HDAC3 is a critical regulator of clock function [50]. NO in turn exerts feed-forward amplification of NF-κB signaling [51]. As multiple clock genes contain NF-κB binding motifs in their promoters, cytokine regulated β-cell clock gene expression could be NF-κB signaling dependent as in other cells, raising the possibility that KDACs and nitroxidative stress mediate cytokine-induced β-cell clock gene expression.

To test these hypotheses, synchronized mouse islets were exposed to IL-1β and IFN-γ, in combination with either a pan-KDAC inhibitor (150 nM givinostat) or the iNOS inhibitor NG-methyl-L-arginine (1 mM NMA). None of these inhibitors prevented cytokine-induced circadian changes (Figure S6A,B), and NMA did not prevent GLT-mediated circadian disruption either (Figure S6C). However, as the cytokines did not decrease viability in the primary islets under the conditions used (Figure 1D), the rescue potential of the inhibitors to influence clock gene expression might depend on their capacity to prevent the cytokine toxicity seen in Figure 3A.

**Figure 7:**
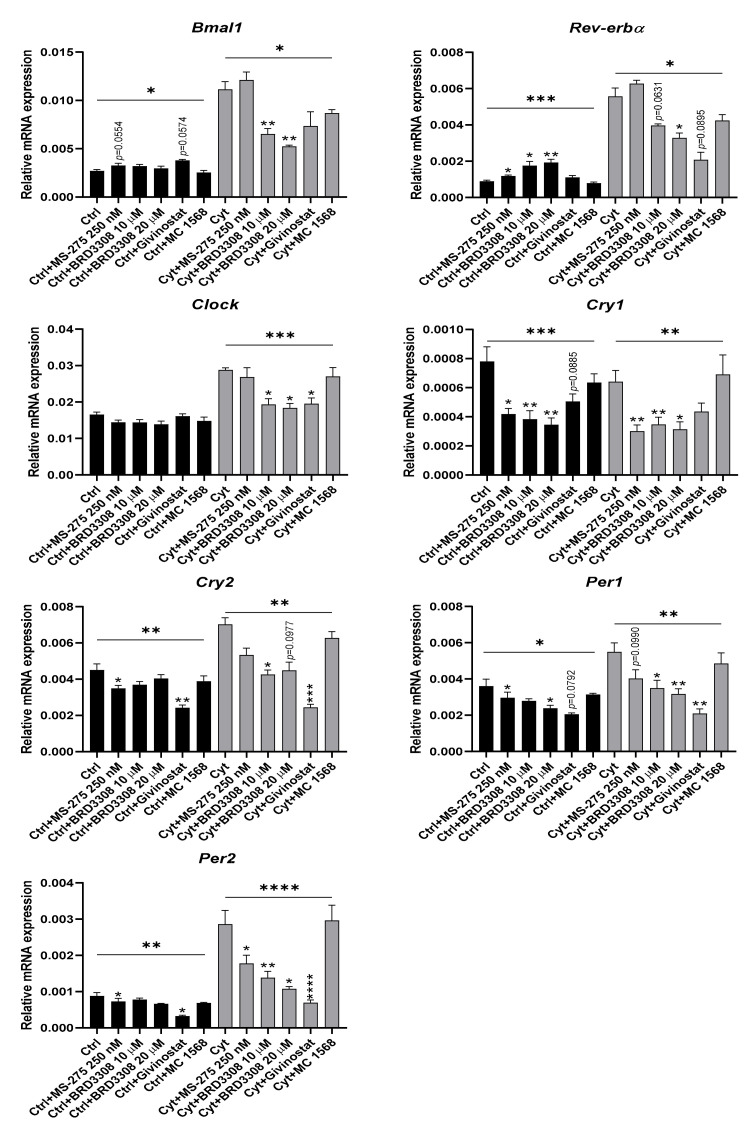
Lysine deacetylase (KDAC) inhibition reduces cytokine-mediated changes in clock gene expression in non-synchronized INS-1 cells. INS-1 cells were treated with 250 nM MS-275, 10 or 20 µM BRD3308, 150 nM givinostat, or 2 µM MC-1568 for 1 h pre-incubation followed by 12 h co-incubation with or without 150 pg/mL mouse IL-1β + 0.1 ng/mL rat IFN-γ (Cyt). Relative mRNA expression is calculated using peptidylprolyl isomerase A (*Ppia*) as reference gene. Values are means ± SEM (*n* = 4). Statistics are one-way ANOVA with *p*-values represented by symbols above the line and with Dunnett’s corrected multiple comparisons to Ctrl (black bars) or to Cyt (gray bars). Significance levels were annotated as follows: * = *p*-value < 0.05, ** = *p*-value < 0.01, *** = *p*-value < 0.001, **** = *p*-value < 0.0001.

Therefore, INS-1 cells were preincubated for one hour with specific inhibitors against class I KDACs (250 nM or 1 µM MS-275), class II KDACs (2 µM MC1568), HDAC3 (10 or 20 µM BRD3308), or a panKDAC inhibitor (givinostat 150 nM), and then exposed for 12 h to IL-1β+IFN-γ in the presence of inhibitors. KDAC inhibition, and in particular inhibition of HDAC3, reduced cytokine-induced clock gene changes, although with varying efficacy and specificity for the investigated gene candidates (Figure 7 and Figure S7).

**Figure 8:**
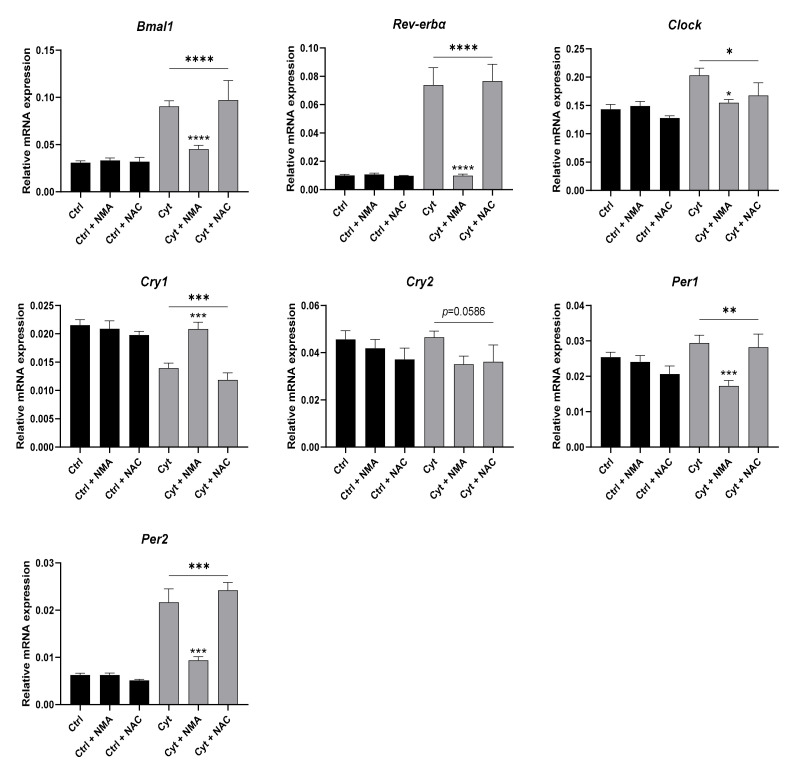
Inhibition of nitroxidative, but not oxidative, stress reverses the cytokine-mediated changes in clock gene expression in non-synchronized INS-1 cells. INS-1 cells were treated with 1 mM NAC or 1 mM NMA for 12 h with or without or without 150 pg/mL mouse IL-1β + 0.1 ng/mL rat IFN-γ (Cyt). Relative mRNA expression is calculated using *Hprt1* and 5S rRNA as reference genes. Values are means ± SEM (*n* = 3–9). Statistics are one-way ANOVA with *p*-values represented by symbols above the line and with Dunnett’s corrected multiple comparisons to Cyt (gray bars). Significance levels were annotated as follows: * = *p*-value < 0.05, ** = *p*-value < 0.01, *** = *p*-value < 0.001, **** = *p*-value < 0.0001.

Having shown that KDACs mediate cytokine-induced upregulation of clock genes, we next explored the effect of nitroxidative stress inhibition. ROS affect NF-κB signaling by either inhibiting or activating the pathway and could thus potentially be involved in this pathway as well. INS-1 cells were exposed to cytokines for 12 h in the absence or presence of the ROS scavenger n-acetylcysteine (NAC) at 5 mM or 1 mM NG-methyl-L-arginine (NMA) (Figure 8). NMA reverted cytokine-induced expression of *Bmal1*, *Rev-erbα*, *Clock*, *Cry1*, and *Per2* (overall trend for *Cry2*), and reduced expression of Per1, while NAC, shown to reduce cytokine induced ROS production (Figure S8), was ineffective. Thus, cytokine-induced clock gene expressional changes were NO-, but not ROS-dependent.

While SR9009 increased ROS levels in INS-1 cells (Figure 5G,H), it did not induce NO formation, but rather reduced it at 5 µM after 24 h exposure (Figure S9A,B). A modest but significant increase in accumulated nitrite was observed at 20 μM in the absence of cytokines (Figure S9A). SR9009 in concentrations associated with toxicity decreased cytokine-induced NO production, even though a concomitant trend towards an increase in *Inos* was observed. The decrease in NO production was not explained by an increase in arginase-1 (*Arg-1*) expression (Figure S9C,D), which would otherwise have led to breakdown of L-arginine as a substrate for iNOS. Interestingly, the expression pattern of *Inos* appeared to mimic the bimodal effect of SR9009 on *Ins-1* expression (Figure 4C), potentially indicating a more general effect of SR9009 on gene transcription. Since NO was measured as accumulated nitrite over 12 and 24 h, the reduction in NO production by SR9009 during cytokine exposure was most likely due to cytotoxicity.

**Figure 9:**
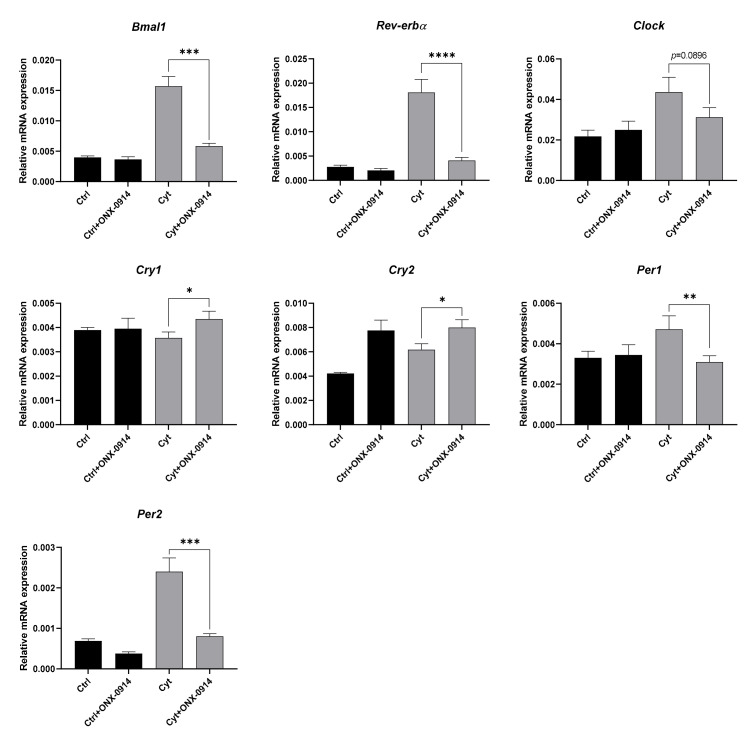
Inhibition of the inducible proteasome reverts cytokine-induced changes in clock gene expression in non-synchronized INS-1 cells. INS-1 cells were treated with 50 nM ONX-0914 for 1 h preincubation followed by 12 h coincubation with or without 150 pg/mL mouse IL-1β + 0.1 ng/mL rat IFN-γ (Cyt). Relative mRNA expression was calculated using *Ppia* as reference gene. Values are means ± SEM (*n* = 5). Statistics are paired Student’s *t*-test. Significance levels were annotated as follows: * = *p*-value < 0.05, ** = *p*-value < 0.01, *** = *p*-value < 0.001, **** = *p*-value < 0.0001.

Proteasomal degradation of clock transcription factors is critical for the circadian transcriptional feedback loop, as demonstrated by stabilization of the BMAL-CLOCK complex upon proteasomal inhibition [52] and degradation of PER and CRY [53]. However, it is not known if the standard proteasome or the immunoproteasome is responsible for degrading clock transcription factors, as inducible subunits, parts of both the intermediate- and immunoproteasome, are constitutively expressed in pancreatic β-cells and upregulated when exposed to IL-1β and IFN-γ [54]. This prompted us to examine if the transcription of clock genes dependent on the BMAL1-CLOCK complex was affected in the context of inflammatory stress when inhibiting the immunoproteasome. Using 50 nM of the inducible subunit PSMB8 inhibitor ONX-0914, the cytokine-mediated effect on *Bmal1*, *Rev-erbα*, *Cry1*, *Per1*, and *Per2* was reversed, while a trend for this effect was observed for *Clock* (Figure 9). However, ONX-0914 further increased cytokine-mediated *Cry2* expression.

Taken together, the effects of cytokine-mediated increase in clock genes appear to be regulated in part by HDAC3, NO, and the immunoproteasome, probably all through NF-κB signaling.

## 3. Discussion

We demonstrated here that cytokines disturb normal circadian behavior in murine and human islets synchronized in vitro, and cause pronounced but uncoordinated increases in mRNA levels of several core clock genes, in particular *Rev-erbα*, in non-synchronized insulin-producing INS-1 cells. The REV-ERBα/β agonist SR9009 reduced the constitutive and cytokine-induced Bmal1 mRNA level in these cells. SR9009 >5 µM induced ROS, reduced *Ins-1/2* mRNA and accumulated, as well as glucose stimulated insulin secretion, reduced cell viability, and increased apoptosis levels in INS-1 cells, thus mimicking cytokine toxicity. SR9009 <5.0 µM increased *Ins-1* mRNA and accumulated but not glucose stimulated insulin secretion without affecting INS-1 cell viability, thus mimicking actions of low, stimulatory cytokine-concentrations. Glucolipotoxic conditions perturbed circadian behavior in synchronized human islets, and significantly reduced *Rev-erbα* and increased *Per1* and *Bmal1* in non-synchronized INS-1E cells associated with ER stress induction, indicating that β-cell oxidative stressors have differential actions on the circadian clock via discrete effectors. Inhibiting NO synthesis, the lysine deacetylase HDAC3 and the immunoproteasome reversed cytokine-induced clock gene expression in INS-1 cells.

The uncoordinated alteration in clock gene expression induced by cytokines in INS-1 cells resembles early induction of several core clock genes by synchronization signals in other cell-types [42]. However, expression of clock repressors and activators in the insulin-producing cell-line was not antiphasic over a 36 h observation period, making it unlikely that cytokine exposure is a synchronization signal in these cells; however, longer observation may be required to reveal antiphasic oscillations. Alternatively, the uncoordinated upregulation of clock genes may be a consequence of IL-1β-induced activation of NF-κB signaling. NF-κB activity is required for functional circadian rhythmicity, and several clock genes harbor NF-κB binding sites [31]. NF-κB activation may also drive the cytokine-mediated perturbation of rhythmicity in synchronized islets in our study. In support of this notion, CLOCK is known to complex with p65 which can repress the expression of clock-controlled genes, and *Clock*-deficient mouse embryonic fibroblasts show reduced NF-κB activity [55,56].

Lipopolysaccharide (LPS)-injected mice display reduced expression in repressor clock genes and upon loss of functional NF-κB signaling; *Per1-3* being increased, while *Bmal1* and *Clock* being decreased in liver [31]. Furthermore, TNFα and IL-1β inhibit clock gene expression in mouse fibroblast in vitro and liver in vivo [57]. This is in contrast to our observation in β-cells that inflammatory stress generally induces the expression of core clock genes in non-synchronized INS-1 cells. This reflects the notable cell-specific difference that NF-κB signaling may exert; in liver tissue, NF-κB is a survival signal, while in β-cells this signal confers functional damage and apoptosis, likely related to differences in signaling duration and kinetics [58] 

The possible involvement of NF-κB signaling in the transcriptional regulation of the β-cell clock is in accordance with our observation that inhibition of KDACs, NO, or the immunoproteasome generally reverts cytokine-mediated alterations in clock gene expression. KDACs and especially HDAC3 are important molecular clock-gene repressors through histone deacetylation [59] or deacetylation-independent actions [50]. Furthermore, CLOCK itself is a lysine acetyltransferase (KAT), acetylating histones in a rhythmic fashion leading to increased transcription, as well as acetylating BMAL1 resulting in recruitment of CRY1 and thus transcriptional repression [60,61]. Inhibition of KDAC activity at the histone level is thus expected to increase expression of core clock genes. However, here KDAC inhibition reverted cytokine-mediated alterations of core clock gene expression, indicating a mode of action independent of histone deacetylation. As we have shown earlier, KDAC inhibition in β-cells promotes hyperacetylation of p65, associated with a decrease in p65 DNA binding and protection from inflammatory stress in vitro and in animal models [47,62], providing a histone-independent mechanism of action of KDAC inhibition on clock gene expression. Indeed, KDACs target more than 4500 non-histone proteins [63].

Oxidative and nitroxidative stress is a hallmark of inflammatory β-cell damage. Here, inhibition of iNOS, but not ROS, reverted cytokine-mediated alterations in clock gene expression. This observation further contributes to the possibility that the observed effect on clock genes during inflammatory stress is NF-κB dependent, as iNOS is a prototypic NF-κB driven gene producing NO harmful for the β-cell [49]. NO exerts a feed-forward effect on NF-κB activity [51,64], and thus by inhibiting NO synthesis, NF-κB signaling is blunted. Alternatively, iNOS inhibition de-represses the transcriptional activity of REV-ERBα as has been shown in other cells [65], which might explain the reduction in cytokine-induced E-box controlled gene expression observed in INS-1 cells.

Inhibition of the inducible proteasome in cytokine-exposed non-synchronized INS-1 cells largely normalized clock gene expression. Both the BMAL1-CLOCK complex and CRYs and PERs are proteasomally degraded, as part of the normal functional clockwork. In pancreatic islets, the inducible proteasome is constitutively expressed, and both IL-1β and IFN-γ further increase expression [54,66]. The transcription of a number of the inducible proteasomal subunits depends on NF-κB signaling, and notably, the inducible proteasome processes NF-κB precursors to active transcription factors [67], further providing a link between NF-κB signaling and cytokine-mediated clock perturbation. Inhibition of proteasomal degradation of both the BMAL1-CLOCK complex and the repressors CRY and PER would be expected to lead to a reduction in the expression of the clock repressor genes, as the transcriptional efficiency of BMAL1-CLOCK would be decreased. This prediction was fulfilled by the observed reduction in *Rev-erbα* and *Per1/2*. However, during inhibition of the inducible proteasome subunit β5i (PSMB8), an increase in the expression of *Cry1/2* was seen, indicating that the effect observed by inhibition of the inducible proteasome during cytokine exposure does not solely depend on the aforementioned proteasomal regulation of the molecular clockwork.

KDAC or iNOS inhibition did not prevent cytokine perturbation of circadian rhythmicity in synchronized rodent reporter islets, similar to the observation that iNOS inhibition failed to affect LPS-induced phase shift in PER2 rhythmicity in mouse macrophages [68]. Since cytokine-exposure did not induce apoptosis in the reporter islets but did modestly reduce viability in the INS-1 cells ~15% in parallel with inducing clock gene expression (Figure 2), we conclude that cytokines alter clock gene expression in the cytotoxic concentration range. Since KDACs and iNOS have been implicated only in the β-cell cytotoxic actions of cytokines, this may account for the failure of KDAC or iNOS inhibition to prevent clock perturbation in synchronized islets in non-toxic culture conditions which may be conferred by the NF-κB regulated non-damaging β-cell transcriptome [69]. In addition, since NO is dispensable as effector of cytokine toxicity in murine and human islets [70,71] it may not be unexpected that KDAC or iNOS inhibition was ineffective.

To clarify the specificity of inflammatory β-cell clock perturbation, we repeated the experiments under glucolipotoxic conditions, known to cause β-cell toxicity mainly via ER stress and ROS dependent upon enhanced iron import [45]. GLT increased period length while delaying the phase in synchronized human reporter islets, as does the application of high glucose alone [19]. Interestingly, GLT caused a clock gene expression profile in non-synchronized INS-1E cells different from that seen during inflammatory stress. Accordingly, oxidative stress induced by H_2_O_2_ induces substantial phase shifts of the Per2-luc rhythmicity in mouse embryonic fibroblasts in vitro and in mouse peripheral tissues in vivo [72]. There is extensive evidence proving ROS formation in cytokine-exposed cells including β-cells and linking ROS formation to the pathological consequences of circadian clock disruption [73,74], and ROS are established effectors of the adverse actions of GLT. Surprisingly and contrary to our anticipation of ROS as a common mechanistic link between cytokine or GLT exposure and circadian clock perturbation, we demonstrated that inhibition of nitroxidative stress, but not oxidative stress, alleviated alterations in cytokine-mediated clock gene expression. Thus, the differential effects of inflammatory and metabolic stress on the β-cell clockwork clock genes may be explained by the observation that inflammatory stress induces both nitroxidative and oxidative stress, while GLT only induces oxidative stress [75]. Accordingly, GLT perturbation of circadian oscillations in reporter islets was unaffected by iNOS inhibition.

We used the REV-ERBα/β agonist SR9009 to mimic the effects of cytokine on *Rev-erbα* mRNA levels to examine the functional consequences of induction of *Rev-erbα* in the absence of potentially confounding cytokine-induced proinflammatory signaling, and found that SR9009 mimicked the bimodal effect of IL-1β on β-cell function and viability. Consistent with previous studies reporting lowered *Bmal1* expression upon SR9009 exposure [76,77], we showed a dose-dependent reduction in *Bmal1* mRNA after 12 and 24 h SR9009 exposure in the absence of cytokines, using concentrations adapted from a recent study in cancer cell lines [78], hereby proving the anticipated biological activity of the agonist. High SR9009 concentrations (>5.0 µM) were shown to reduce cell viability, increase apoptosis and to abrogate both accumulated and stimulated insulin release, associated with markedly reduced *Bmal1* and diminished mRNA levels of both *Ins1/2* and increase in ROS production, in accordance with the observation that increased Bmal1 level by either β-cell specific overexpression or ROR-induction improves β-cell secretory function in vitro and in vivo [79]. In remarkable contrast, low concentrations of SR9009 (<5.0 µM) reduced *Bmal1* but increased *Ins1* mRNA levels and increased accumulated insulin release but not GSIS without affecting cell viability, indicating a beneficial effect on insulin biosynthesis at low levels of *Bmal1* inhibition. To further explore the dynamic effect of SR9009 on insulin secretion, future experiments using islets perifusion should be performed. The enhancing effect on insulin secretion at SR9009 concentrations where ROS production starts to increase without affecting GSIS, and the reduction in insulin secretion at high levels of ROS is consistent with the observation that ROS increases basal insulin secretion [44], while excessive ROS production blunts insulin secretion [80]. Studies in human and rodent islets [18,81,82] have shown that a functional β-cell clock is required for appropriate basal and stimulated insulin secretion, and that clock perturbation has a pronounced impact on essential genes involved in insulin secretion. Specifically, Clock knockdown by specific small interfering RNA (siRNA) revealed no alterations in expression of the insulin gene itself, whereas several genes crucial for granule formation, membrane fusion, and intracellular signal transduction related to insulin synthesis and secretion were downregulated in human islets [18]. Interestingly, human islets derived from type 2 diabetes individuals exhibited attenuated circadian oscillations, paralleled with reduced insulin and glucagon exocytosis [19]. SR9009 has shown promising effects in diet-induced obese mice including increased energy expenditure and reduced levels of fat mass, plasma triglycerides, and cholesterol [83]. Additionally, instead of causing complete disruption of nocturnal locomotor activity, SR9009 exposure induced a 1–3 h delay in the locomotor activity in mice, thereby indicating a potential role of SR9009 in treating both metabolic diseases and sleep disorders [83,84]. We further demonstrate that SR9009 treatment in presence of cytokines did not sensitize INS-1 cells to cytotoxicity. It is possible that protective and/or sensitizing actions of SR9009 are masked by an overshadowing influence of proinflammatory signals induced by the concentrations of cytokines used, and experiments with titration of cytokine concentrations at low SR9009 should be performed.

A caveat in the interpretation of the results obtained with high concentrations of SR9009 is that this compound may reduce cell viability and mitochondrial function in a REV-ERBα/β independent fashion [85]. To definitively rule out the possibility of idiosyncrasy and off-target effects by the REV-ERBα/β agonist, molecular approaches such as knockdown of one or more core clock genes in a β-cell or animal model by either siRNA or CRISPR-Cas9 should be applied. To further investigate the mechanisms of proinflammatory cytokine action on clock gene expression, knockdown of different elements of regulatory pathways should be undertaken, as the main findings in this study are derived from pharmacological inhibition. We hypothesize that NF-κB plays a key role in cytokine-mediated clockwork alterations. However, the precise molecular regulation of NF-κB transcriptional activity conferred by KDACs, NO and the inducible proteasome have to be further explored. Lastly, the mechanism of action leading to alteration in clock gene expression under metabolic stress needs to be addressed, potentially focusing on ROS and ER stress pathways.

IL-1β was recently reported to reduce *Bmal1* mRNA and protein levels associated with a decrease in RORα and an increase in REV-ERBα protein levels, while TNFα and IL-6 reduced *Bmal1* mRNA expression, and IL-6 and IFN-γ reduced *Clock* mRNA expression in INS-1 832/13 cells [40]. Only a limited set of core clock genes was examined, whereas we profiled a more extensive set of core clock genes. In addition, the INS-1 832/13 cells [40] were exposed to cytokines for twice as long as the cells of our study. As shown in Figure S2A, β-cell core clock gene expression is time dependent, and long exposure enhances the risk of observing effects secondary to cytokine toxicity including apoptosis, which was not investigated in [40]. Further, the synergistic effects of the Type 1 diabetes relevant combination of cytokines, IL-1β + IFN-γ was not investigated, and tracing of circadian oscillations in human reporter islets were not done to probe the human translational potential of the observations obtained with the murine reporter system.

We conclude from these studies that inflammatory and metabolic stress perturb the intrinsic β-cell clock depending on cytokine concentrations and exposure time, stress stimulus as well as cellular model system. The uncoordinated cytokine-mediated induction of clock gene expression in non-synchronized INS-1 cells depended in part on NO, HDAC3, and immunoproteasome activity, however, the exact mode of action remains to be explored. Clock perturbation by high concentrations of the REV-ERBα/β agonist mimicked cytokine toxicity but did not sensitize insulin-producing cells to cytokines. Low concentrations of REV-ERBα/β agonist may increase INS-1 cell insulin mRNA levels and constitutive insulin secretion, compatible with a beneficial effect on insulin biosynthesis. However, more studies are needed to confirm these findings compatible with an advantageous effect on insulin biosynthesis. Further research exploring the mechanisms of action and importance of clock disruption by cytokines in vivo and in vitro (including studies on both primary murine and human islets, and the EndoC-bH3 cell line) is needed to judge the relevance of these findings for the future development of antidiabetic drugs.

## 4. Materials and Methods

### 4.1. Human Pancreatic Islets

Human pancreatic islets were obtained from two different sources: (i) Prodo Laboratories LTD company and (ii) Islet Transplantation Center of Geneva University Hospital. Details of the islet donors are summarized in Table S1. The experiments were conducted on human islets that were cultured in CMRL 1066 (ICN Biomedicals, Costa Mesa, CA, USA) medium, supplemented with 10% fetal bovine serum, 2 mM L-glutamine, 50 μg/mL gentamycin, 110 U/mL penicillin, and 110 μg/mL streptomycin at 37 °C and 5% CO_2_ up to two weeks, as described in [19]. All procedures using human islets were approved by the ethical committee of Geneva University Hospital (CCER 2017-00147, 21 February 2017).

### 4.2. Lentiviral Transduction of Human Islets

To produce lentiviral particles, Per2-luc lentivectors were transfected into 293T cells using the polyethylenimine method as described in detail in [86]. High efficiency of human islets transduction by lentiviral vectors was previously confirmed by us in intact human islets using co-transduction of luciferase reporter particles (Bmal-Luc) with the fluorescent reporter construct CMV-GFP, with a multiplicity of infection of three [86].

### 4.3. Animal Care, Mouse Strain, and Pancreatic islet Preparations

Animal studies were performed according to the regulations of the veterinary office of the State of Geneva (authorization number GE/159/15, 28 March 2019). The Triple reporter mouse line *proGcg*-Venus/RIP-Cherry/PER2:Luc was used for the islet isolation experiments [87]. All the experiments were done in both male and female mice aged 7–16 weeks, under standard animal housing conditions with ad libitum access to food and water and 12 h light/12 h dark cycles (LD). Islets of Langerhans were isolated based on collagenase (Type XI, Sigma, Saint-Louis, MO, USA) digestion of pancreas followed by Ficoll purification, as described in detail in [87].

### 4.4. In Vitro Islet Synchronization and Circadian Bioluminescence Recording

Adherent islets were synchronized by a 1-h pulse of forskolin (10 μM; Sigma) with a subsequent medium change. The islets were subjected to continuous bioluminescence recording in CMRL (human islets) or RPMI (mouse islets) medium containing 100 μM luciferin (D-luciferin 306-250, NanoLight Technology, Pinetop, AZ, USA) during several days. The experimental duration was as indicated in the respective graphs. For the experiments with proinflammatory cytokines and glucolipotoxicity (GLT) treatment, the following agents were added to the forskolin mixture at the time of synchronization and added to the recording medium for the entire experimental duration: (i) 300 pg/mL or (ii) 2 ng/mL of IL-1β; (iii) combination of 300 pg/mL IL-1β and 0.2 ng/mL IFN-γ (cyt); (iv) 500 nM palmitate diluted in bovine serum albumin (BSA, Sigma) at a molar ratio 6:1 and 20 mmol glucose; (v) BSA; (vi) 20 mmol glucose. Lysine deacetylase inhibitor Givinostat (Sigma), 150 mM, or 1 mM iNOS inhibitor NG-methyl-L-arginine (NMA, Sigma) was applied to the islets starting from the synchronization step and added during the recording, where indicated. Bioluminescence pattern was monitored as described previously [87]. The amplitude, period length, and circadian phase were analyzed based on the processed data representing a moving average with a window of 24 h (detrended values, [18]). Average circadian amplitude, period length, and phase were calculated based on 4 consecutive peaks of detrended profiles starting from the second circadian cycle. Cell apoptosis was assessed with Cell Death Detection ELISA^PLUS^ kit (Roche, Mannheim, Germany) at the end of bioluminescence experiments, where indicated, according to manufacturer instructions.

### 4.5. Cell Culture

The rat INS-1 and INS-1E insulinoma cell lines were kindly provided by C. Wollheim and P. Maechler, University Medical Centre, Geneva, Switzerland, and were precultured and cultured as in [88]. Following pre-culture, cells were exposed to cytokines (150 pg/mL IL-1β and 0.1 ng IFN-γ as standard conditions, or as specified in figure legends) or, with the aim of testing the specificity of the effects of cytokines relative to other inducers of β-cell oxidative stress, to a glucolipotoxic condition consisting of 0.5 mM palmitate conjugated with 1% endotoxin-free bovine serum albumin at a molar ratio of 3.33:1 + 25 mM glucose, as specified in the figure legends.

### 4.6. RT-qPCR

One million cells/well were seeded in six-well plates and total RNA was extracted using NucleoSpin^®^ RNA kit (Macherey-Nagel, Bethlehem, PA, USA), followed by assessment of quantity and purity of the extracted RNA using a NanoDrop-2000 (Thermo Scientific, Copenhagen, Denmark). cDNA was synthesized from 500 ng RNA using the iScript™-cDNA Kit (BioRad, Copenhagen, Denmark). Gene expression was quantified by real-time reverse transcriptase-quantitative PCR (RT-qPCR) using gene-specific primers (Table S2) with SYBR^TM^ Green PCR Master Mix (Applied-Biosystems, Naerum, Denmark) using the 7900HT Fast Real-Time PCR System (Applied-Biosystems) or QuantStudio™ 5 (Applied-Biosystems). All reactions were carried out in accordance to the manufacturers’ protocols. Relative mRNA expression was determined, and statistical analysis was carried using ΔCt values (Ct_target gene_-Ct_reference gene_), while visualized by the 2^-ΔCt^ method. Inter- and intragroup variation of potential reference genes were evaluated using the NormFinder software [89], and by manually evaluating the stability of the raw Ct values. From this, the most stable expressed reference gene was selected in each experiment to provide the most accurate normalization. In cases where the combination of two reference genes displayed the most stable expression profile, the geometric mean of the two was used for normalization.

### 4.7. Nitric Oxide (NO) Assay

Accumulated nitrite from cell supernatants was quantified by the Griess reagent assay as a proxy for NO production as described in [90].

### 4.8. Insulin Secretion Assay

Insulin secretion was assessed by either accumulated insulin in supernatants or glucose-stimulated insulin secretion (GSIS). For GSIS, 300,000 INS-1 cells/well (12-well plate) were pre-incubated for 72 h before being exposed to treatment conditions for 24 h. Medium was subsequently removed and cells were incubated in Krebs-Ringer’s-bicarbonate-Hepes (KRBH) buffer (149 mM NaCl, 1.32 mM NaH_2_PO_4_(H_2_O), 4.4 mM KCl, 2.75 mM CaCl_2_, 1.32 mM MgCl_2_, 25 mM NaHCO_3_, 10 mM HEPES (pH 7.4), and 0.1 % BSA) with 2 mM glucose for two hours. The buffer was removed and new KRBH buffer with 2 mM glucose was added, followed by 16.7 mM glucose for a one-hour incubation at each concentration, and samples were collected at both steps. Insulin was measured using a competitive ELISA as described in [91]. Color reaction was induced using 1-Step™ Ultra TMB-ELISA Substrate Solution (Thermo Scientific).

### 4.9. Apoptosis and Cell Viability Assays

Fifty thousand INS-1 cells/well (48-well plate) were precultured for 72 h, before being exposed to cytokines as described in the legends. Apoptosis levels were measured by detection of DNA/histone complexes released to the cytosol using the Roche Cell Death Detection ELISA^PLUS^ (Roche, Mannheim, Germany). INS-1 cells (30,000 per well in a 96-well plate) were used to determine viability/mitochondrial function by AlamarBlue (Invitrogen, Naerum, Denmark) or MTT (3-(4,5-dimethylthiazol-2-yl)-2,5-diphenyltetrazolium-bromide) (Invitrogen) assays following the manufactures’ instructions.

### 4.10. Reactive Oxygen Species (ROS) Assay

Thirty thousand INS-1 cells/well were precultured in 96-black-walled plates before exposure. CM-H_2_DCFDA (Invitrogen) was used to measure ROS levels (Ex = 492, Em = 527), while ROS-ID^®^ Total ROS/Superoxide detection kit (Enzo, Farmingdale, NY, USA) was used to measure ROS levels excluding superoxide (Ex = 488, Em = 520) or to measure superoxide specifically (Ex = 550 nm, Em = 620 nm) both following the manufacturers’ description. Fluorescence was measured on a SpectraMax^®^ i3 plate reader (Molecular Devices, San Jose, CA, USA).

### 4.11. Statistical Analysis

Statistical analysis and visualization were performed using GraphPad Prism 9, using one-way ANOVA followed by Tukey, Bonferroni, or Dunnett multiple comparisons correction test as indicated in the figure legends or paired post hoc Student’s t-tests where appropriate. Data are presented as mean or mean ± SEM. *p*-values < 0.05 were regarded as significant and <0.1 as a trend. Significance levels were annotated as follows: * = *p*-value < 0.05, ** = *p*-value < 0.01, *** = *p*-value < 0.001, **** = *p*-value < 0.0001.

### 4.12. Inhibitor and Agonist Details

The synonyms, chemical nomenclature, and source of the different inhibitors and agonists are described in Table S3.

## Data Availability

The data presented in this study are available in the present article and supplementary material.

## Supplementary Materials

The following are available online at https://www.mdpi.com/1422-0067/22/1/83/s1.

## Abbreviations

Arg-1	Arginase-1
Bmal1	Brain and muscle arnt-like 1
Chop	C/EBP homologous protein
Clock	Circadian locomotor output cycles kaput
Cry	Cryptochrome
ECIT	European Consortium for islet transplantation
GLT	Glucolipotoxicity
GSIS	Glucose-stimulated insulin secretion
HDAC3	Histone deacetylase 3
Hprt1	Hypoxanthine-guanine phosphoribosyltransferase
IFN-γ	Interferon-γ
IL-1β	Interleukin-1β
IL-6	Interleukin 6
Inos	Inducible nitric oxide synthase
Ins-1/2	Insulin 1/2
IκBα	Nuclear factor of kappa light polypeptide gene enhancer in B-cells inhibitor α
KAT	Lysine acetyltransferase
KDAC	Lysine deacetylase
LPS	Lipopolysaccharide
MAPK	Mitogen-activated protein kinase
MTT	3-(4,5-dimethylthiazol-2-yl)-2,5-diphenyltetrazolium-bromide
NAC	n-acetylcysteine
NF-κB	Nuclear factor of kappa light polypeptide gene enhancer in B-cells
NMA	NG-methyl-L-arginine
NO	Nitric oxide
Per	Period
Per2-luc	Per2-luciferase
Ppia	Peptidylprolyl isomerase A
PSMB8	Inducible proteasome subunit β5i
Rev-erb	Reverse-erythroblastosis virus
RORα	Retinoic acid receptor-related orphan receptor α
ROS	Reactive oxygen species
SCN	Suprachiasmatic nucleus
SI	Stimulatory index
siRNA	Small interfering RNA
TNFα	Tumor necrosis factor α
