# Supplementary material for: Proinflammatory Cytokines Perturb Mouse and Human Pancreatic Islet Circadian Rhythmicity and Induce Uncoordinated β-Cell Clock Gene Expression via Nitric Oxide, Lysine Deacetylases, and Immunoproteasomal Activity
Source: Int J Mol Sci. 2020 Dec 23;22(1):83. doi: 10.3390/ijms22010083 (PMC7795908; doi:10.3390/ijms22010083)
Supplement: Supplementary file 1 [file ijms-22-00083-s001.pdf]

International Journal of *Molecular Sciences*

MDPI

Supplementary Material

Proinflammatory Cytokines Perturb Mouse and Human Pancreatic Islet Circadian Rhythmicity and Induce Uncoordinated β-Cell Clock Gene Expression via Nitric Oxide, Lysine Deacetylases, and Immunoproteasomal Activity

Phillip Alexander Keller Andersen [1,†], Volodymyr Petrenko [2,†], Peter Horskjær Rose [1], Melissa Koomen [1,‡], Nico Fischer [1,‡], Seyed Mojtaba Ghiasi [1], Tina Dahlby [1], Charna Dibner [2,§] and Thomas Mandrup-Poulsen [1,*,§]

1 Department of Biomedical Sciences, Faculty of Health and Medical Sciences, University of Copenhagen, 3 Blegdamsvej, DK-2200 Copenhagen N, Denmark; phillip.andersen@sund.ku.dk (P.A.K.A.); horskjaerrose@gmail.com (P.H.R.); s.ghiasi@imperial.ac.uk (S.M.G.); tina.dahlby@hest.ethz.ch (T.D.); melissa_koomen@hotmail.com (M.K.); nico.fischer98@hotmail.com (N.F.)

2 Division of Endocrinology, Diabetes, Nutrition and Patient Education, Department of Cell Physiology and Metabolism, Diabetes Center, Faculty of Medicine, University of Geneva, D05.2147c Rue Michel-Servet, 1 CH-1211 Geneva 4, Switzerland; volodymyr.petrenko@unige.ch (V.P.); charna.dibner@hcuge.ch (C.D.)

* Correspondence: tmpo@sund.ku.dk

† These authors contributed equally to this work

‡ These authors contributed equally to this work

§ These authors contributed equally to this work

Table S1. Human islet donor characteristics.

Donor no.	Sex	Age (years)	BMI (kg/m²)
1[1]	M	46	27.2
2[2]	M	66	27.0
3[2]	M	25	27.3
4[1]	F	55	21.9

M, male sex; F, female sex.[1]Donor provided by Islet Transplantation Center of Geneva University Hospital;[2]Donors provided by Prodo Laboratories (California, US).

Copyright: © 2020 by the authors. Licensee MDPI, Basel, Switzerland. This article is an open access article distributed under the terms and conditions of the Creative Commons Attribution (CC BY) license (http://creativecommons.org/licenses/by/4.0/).

Table S2. Primer sequences.

Target gene	*Primer sequence*

5S rRNA	*Forward*	5'-TCTTTGGGAAATGGAGCACT-3'
	Reverse	5'-ATGAGCTTCTTGCCGTTGTT-3'
Arg-1	*Forward*	5'-TATCGGAGCGCCTTTCTCTA-3'
	Reverse	5'-ACAGACCGTGGGTTCTTCAC-3'
β-Actin	*Forward*	5'-CACCCGCGAGTACAACCTTC-3'
	Reverse	5'-CCCATACCCACCATCACACC-3'
Bmal1	*Forward*	5'-TAAACTCACCGTGCTCAGGA-3'
	Reverse	5'-CGGTCACATCCTACGACAAA-3'
Chop	*Forward*	5'-CAGCGACAGAGCCAAAATAAC-3'
	Reverse	5'-TGTGGTGGTGTATGAAGATGC-3'
Clock	*Forward*	5'-ACTATACAGCGCACACACAGG-3'
	Reverse	5'-TGTGAACTCTTCATTCGGTTCT-3'
Cry1	*Forward*	5'-CCTTGCCTCAGTCCCTTCTA-3'
	Reverse	5'-GTGCGTCCTCTTCCTGACTT-3'
Cry2	*Forward*	5'-CCTCTTCTACTACCGCCTGTG-3'
	Reverse	5'-ATTCTCGCCATAGGAGTTGTC-3'
Fas	*Forward*	5′-TGAGGGTTTGGAGTTGAAGAG-3′
	Reverse	5′-CCACTTGTTGTGCAGTCCTTA-3′
Hprt1	*Forward*	5'-GCAGACTTTGCTTTCCTT-3'
	Reverse	5'-CCGCTGTCTTTTAGGCTT-3'
Inos	*Forward*	5'-CACCACCCTCCTTGTTCAACA-3'
	Reverse	5'-CAATCCACAACTCGCTCCAA-3'
Ins-1	*Forward*	5'-GGGGAACGTGGTTTCTTCTAC-3'
	Reverse	5'-CCAGTTGGTAGAGGGAGCAG-3'
Ins-2	*Forward*	5'-CAGCACCTTTGTGGTTCTCA-3'
	Reverse	5'-CACCTCCAGTGCCAAGGT-3'
Nfkbia (IκBα)	*Forward*	5′-ATTACGAGCAGATGGTGAAGG-3′
	Reverse	5′-GGTCAGTGTCTTCTCTTCATGG-3′
Per1	*Forward*	5'-GCTCCATTGCCTATAGTCTCCT-3'
	Reverse	5'-AAGTGCGGTCATGAGTTCTTT-3'
Per2	*Forward*	5'-GTGACTGTGACGACAGTGGAA-3'
	Reverse	5'-CTTGTGGAGGGGTTATGCTC-3'
Ppia	*Forward*	5'-AGCACTGGGGAGAAAGGATT-3'
	Reverse	5'-GATGCCAGGACCTGTATGCT-3'
Rev-erbα	*Forward*	5'-TTTGGACGTATCCCCAAGAG-3'
	Reverse	5'-CTGAGAGAAGCCCACCAAAG-3'

Table S3. Inhibitor and agonist details.

**Compound**	**Synonym**	**Chemical nomenclature**	**Manufacture**

BRD3308		4-Acetamido-N-(2-amino-4-fluorophenyl)benzamide	Sigma
Givinostat	ITF2357	N-[4-[(Hydroxyamino)carbonyl]phenyl]-carbamic acid	Sigma
MC 1568		3-[5-(3-(3-Fluorophenyl)-3-oxopropen-1-yl)-1-methyl-1H-pyrrol-2-yl]-N-hydroxy-2-propenamide	Tocris, Abingdon, UK
MS-275	Entinostat	(Pyridin-3-yl)methyl 4-(2-aminophenylcarbamoyl)benzylcarbamate	Tocris
NAC		N-Acetyl-L-cysteine	Sigma
NMA		NG-Methyl-L-arginine acetate salt	Sigma
ONX 0914	PR-957	N-[2-(4-morpholinyl)acetyl]-L-alanyl-O-methyl-N-[(1S)-2-[(2R)-2-methyl-2-oxiranyl]-2-oxo-1-(phenylmethyl)ethyl]-L-tyrosinamide	Cayman Chemical, Ann Arbor, Michigan, USA
SR9009		Ethyl 3-[[[(4-chlorophenyl)methyl][(5-nitro-2-thienyl)methyl]amino]methyl]-1-pyrrolidinecarboxylate	Tocris

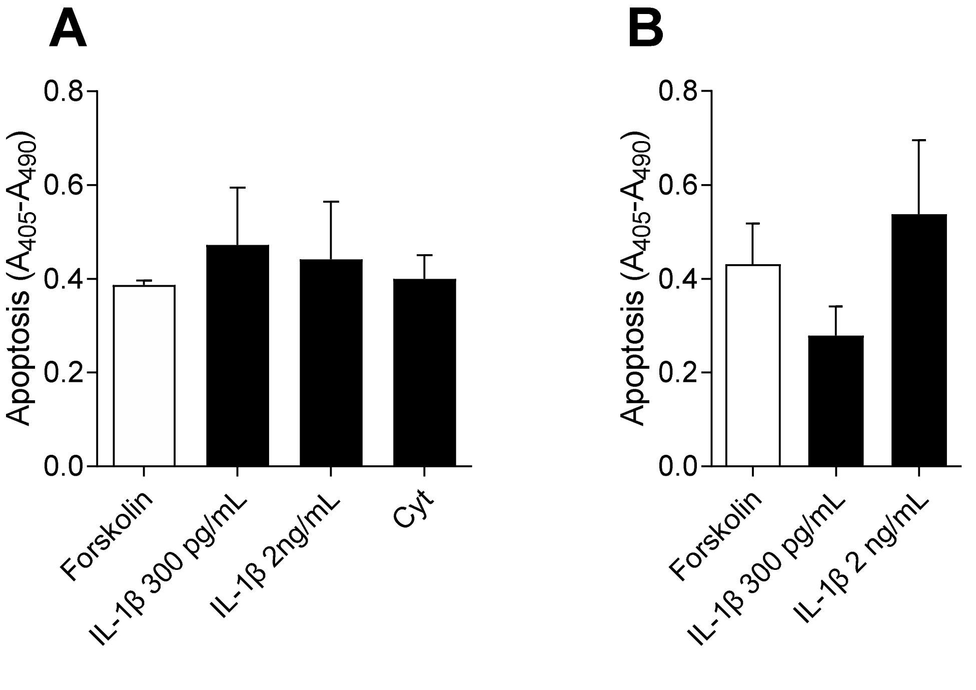

Figure S1. Cytokines do not cause apoptosis in synchronized mouse or human Per2-luc reporter islets. Islet cell apoptosis was measured using Cell Death ELISA Kit (Roche) at the end of the

bioluminescence recording experiments from mouse (**A**, n = 2 islet isolations with 2-3 animals per isolation) and human (**B**, n = 3 human donors) islets. Values are means ± SEM.

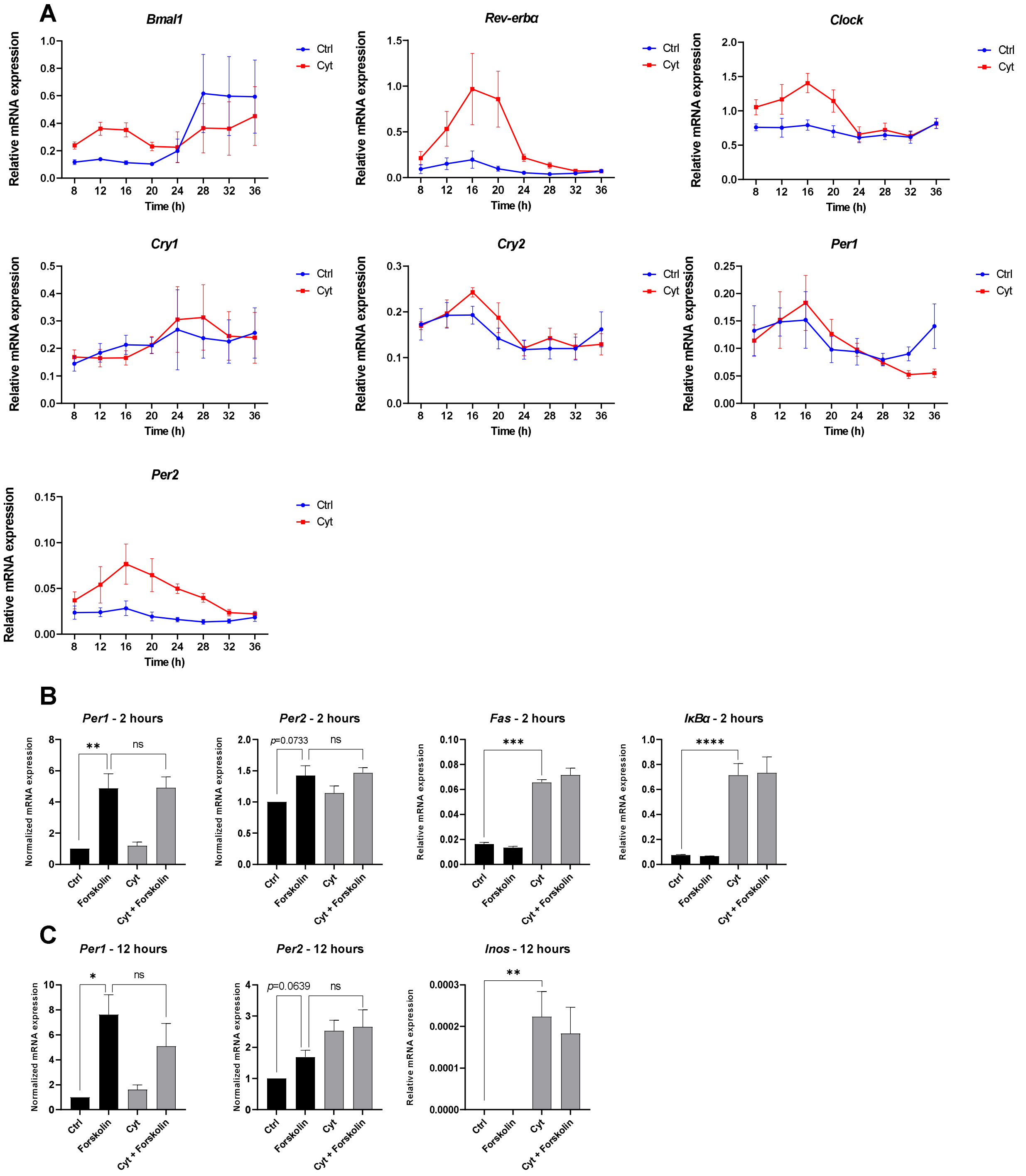

Figure S2. Time-dependent differential responses in clock gene expression to proinflammatory cytokines in non-synchronized INS-1 cells and lack of effects of cytokines on response to forskolin. (A) INS-1 cells were exposed to 150 pg/mL mouse IL-1β + 0.1 ng/mL rat IFN-γ (Cyt) at 4 hours intervals at 8-36 hours. Relative mRNA expression is calculated using *Hprt1* as reference gene (n = 5). (B, C) INS-1 cells were treated with 10 μM forskolin, for either one-hour pulse with or without cytokines, following one-hour exposure to control media or cytokines (B) or 12-hours preincubation with cytokines following one-hour forskolin pulse in normal media (C). Samples were collected one hour after the one-hour forskolin pulse. Relative mRNA expression is calculated using *Hprt1* and 5S rRNA as reference genes (n = 3-4). Values are means ± SEM. Statistics are paired Student's t-test. ns:

not significant. Significance levels were annotated as follows: *= p-value< 0.05, **= p-value< 0.01, ***= p-value < 0.001, ****= p-value < 0.0001.

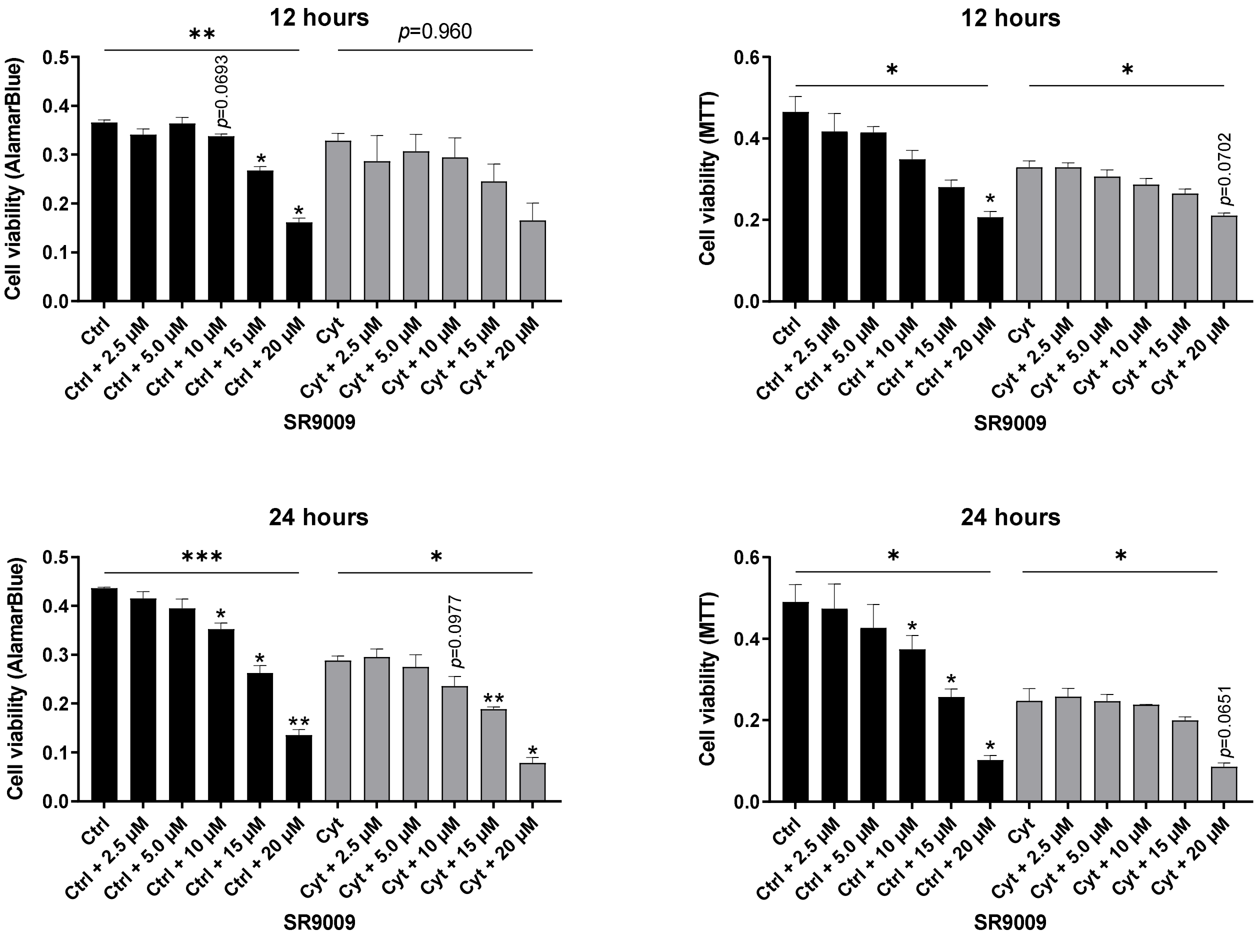

Figure S3. SR9009 does not sensitize non-synchronized INS-1 cells to cytokine-mediated cytotoxicity. INS-1 cells were exposed to increasing concentrations of SR9009 in combination with 150 pg/mL mouse IL-1β + 0.1 ng/mL rat IFN-γ (Cyt) for 12 or 24 hours to assess the effect on cell viability. Values are means ± SEM (n = 3). Statistics are one-way ANOVA with p-values represented by symbols above the line and with Dunnett's corrected multiple comparisons to Ctrl (black bars) or to Cyt (grey bars). Significance levels were annotated as follows: *= p-value< 0.05, **= p-value< 0.01, ***= p-value < 0.001.

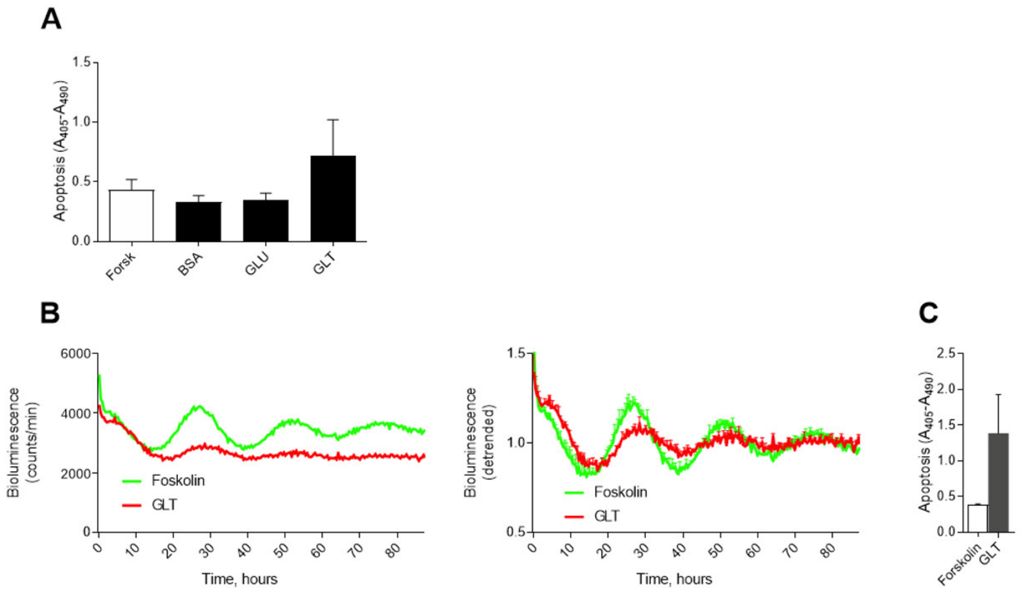

Figure 4. Glucolipotoxicity increases apoptosis and perturb the clockwork in human or mouse Per2-luc reporter islets. (A) Levels of apoptosis in human islets (n = 3) exposed to a combination of 500 nM palmitate and 20 mmol glucose (GLT); to 20 mmol glucose (GLU), or to BSA alone, were measured by Cell Death ELISA Kit (Roche) at the end of the bioluminescence recording experiments. (B) Average raw (left panel) and detrended (right panel) Per2-luc oscillatory profiles of forskolin-synchronized mouse islets (n = 2 independent isolations from 2-3 animals each) exposed to GLT. (C) Levels of apoptosis in mouse islets (n = 2 experiments). Values are means or means ± SEM.

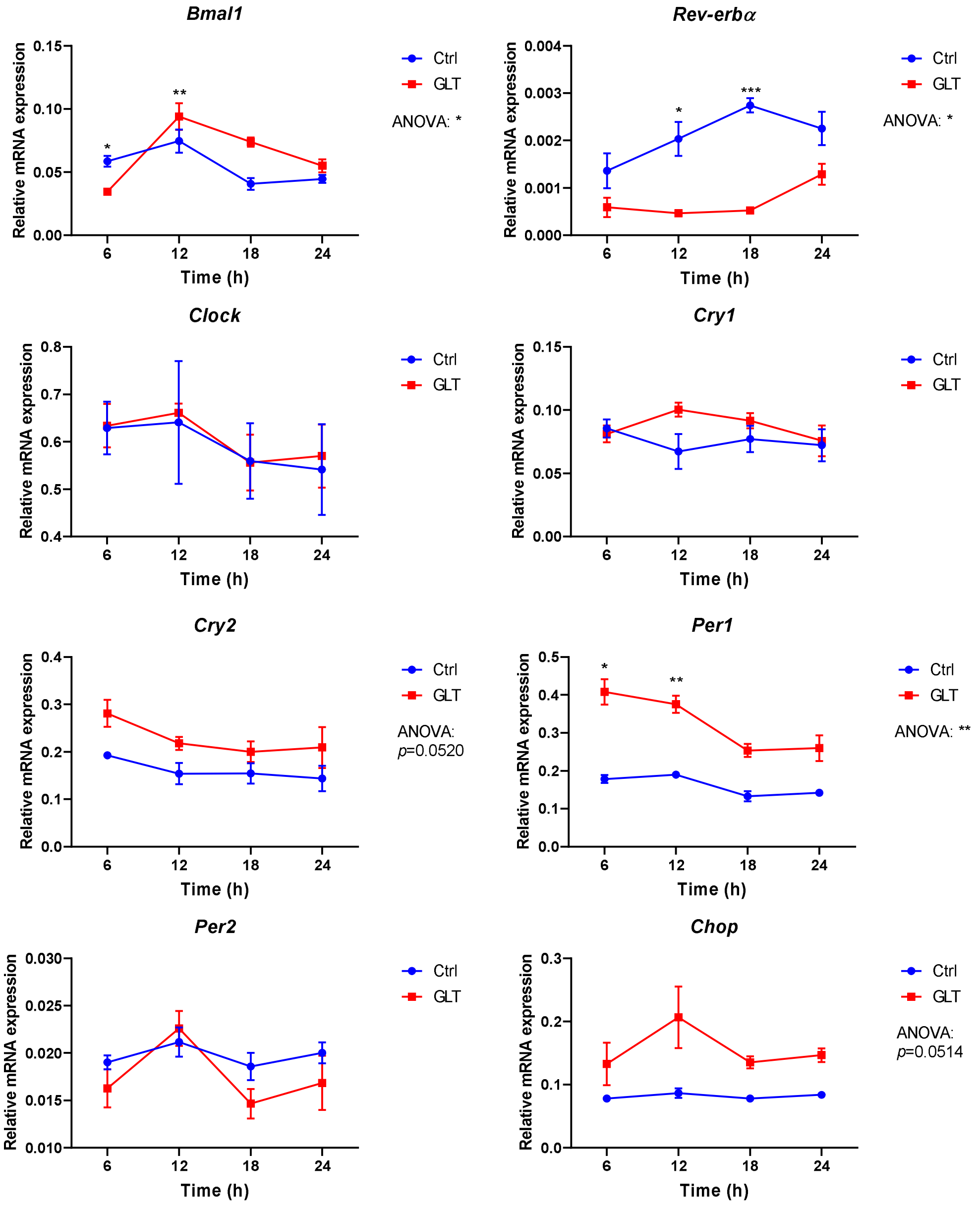

Figure S5. Glucolipotoxicity time-dependently and differentially affects clock gene expression in non-synchronized INS-1E cells. INS-1E cells were exposed to 0.5 mM palmitate+ 25mM glucose (GLT) for 6, 12, 18 or 24 hours. Relative mRNA expression is calculated using *Hprt1* as reference gene. Values are means ± SEM (n = 4). Statistics are repeated measures one-way ANOVA with Bonferroni corrected multiple comparisons between Ctrl and GLT at similar timepoints. Significance levels were annotated as follows: *= *p*-value< 0.05, **= *p*-value< 0.01, ***= *p*-value < 0.001.

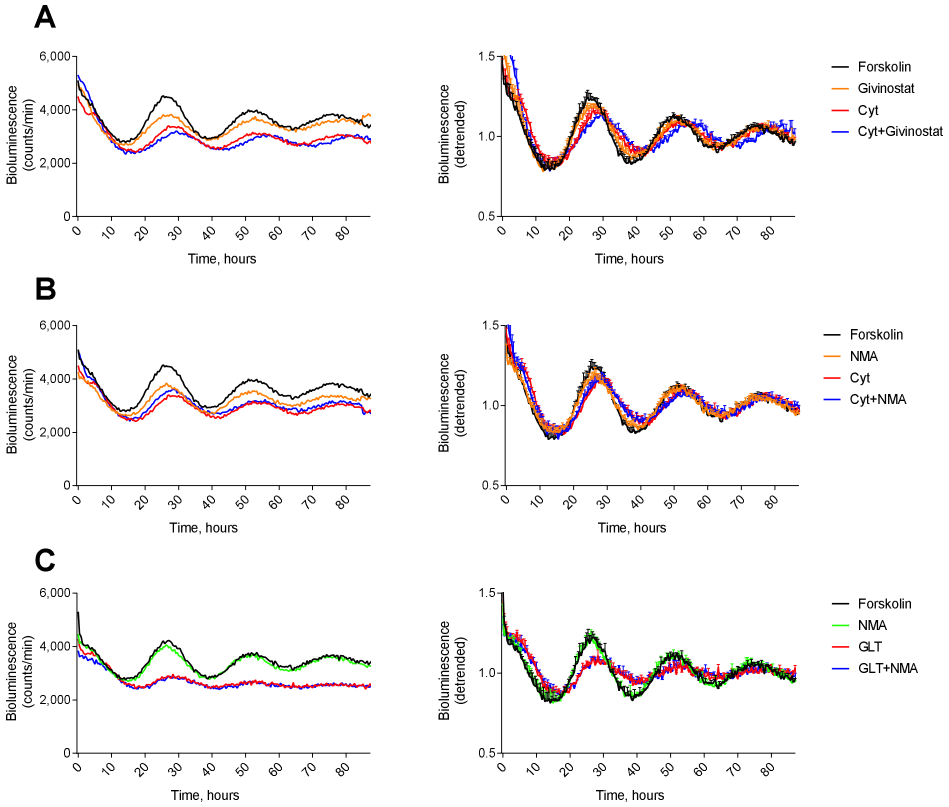

Figure S6. iNOS or KDAC inhibition does not affect cytokine-perturbation of the clockwork in synchronized mouse Per2-luc reporter islets. (A-B) Average raw (left panel) and detrended (right panel) Per2-luc oscillatory profiles of forskolin-synchronized mouse islets exposed to a combination of 300 pg/mL IL-1β + 0.2 ng/mL IFN-γ (Cyt) in the presence of Givinostat (A, n = 3 experiments) or iNOS inhibitor NG-methyl-L-arginine (NMA, B, n = 3 experiments) during the entire recording. (C) Average raw (left panel) and detrended (right panel) Per2:Luc bioluminescence profiles of forskolin-synchronized mouse islets exposed to GLT in the presence or absence of NMA during the entire recording (n = 2 experiments). Values are means or means ± SEM.

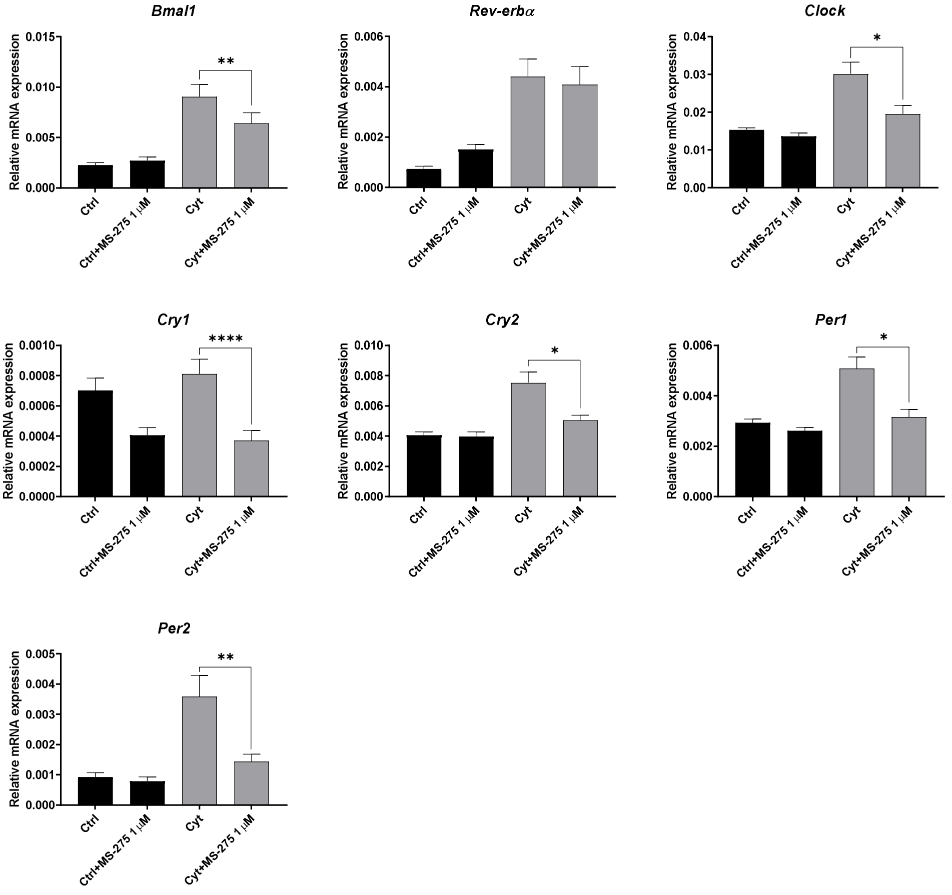

Figure S7. Class I KDAC inhibition reduces cytokine mediated changes in clock gene expression in non-synchronized INS-1 cells. INS-1 cells were treated with 1 μM MS-275 for 1 hour pre-incubation followed by 12 hour co-incubation with or without 150 pg/mL mouse IL-1β + 0.1 ng/mL rat IFN-γ (Cyt). Relative mRNA expression is calculated using *Ppia* as reference gene. Values are means ± SEM (n = 6). Statistics are paired Student's t-test. Significance levels were annotated as follows: *= *p*-value< 0.05, **= *p*-value< 0.01, ****= *p*-value < 0.0001.

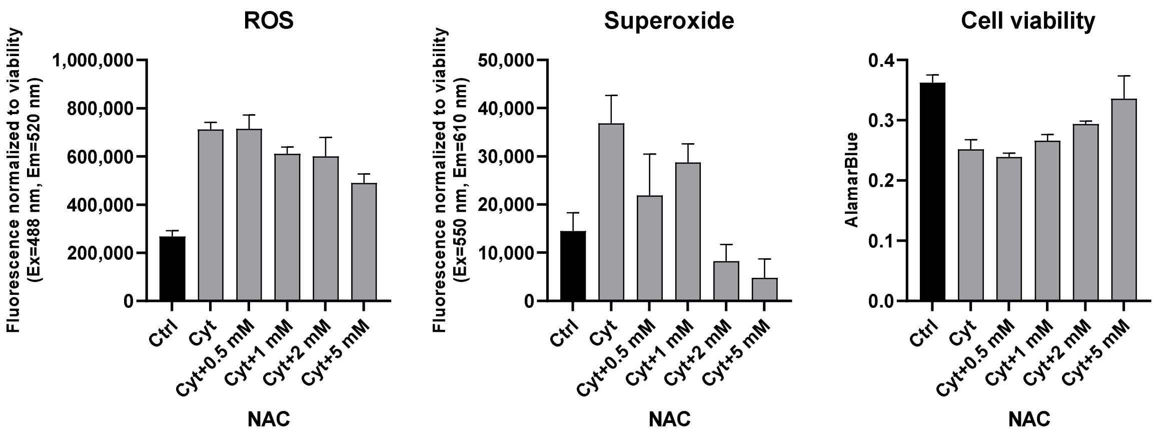

Figure S8. NAC dose-dependently lowers ROS and superoxide production in INS-1 cells. INS-1 cells were treated with different doses of NAC for 12 hours with or without or without 150 pg/mL mouse IL-1β + 0.1 ng/mL rat IFN-γ (Cyt). Florescent values from the two probes using ROS-ID® Total ROS/Superoxide detection kit are normalized to the viability of the cells. Values are means ± SEM of 5-6 technical replicates.

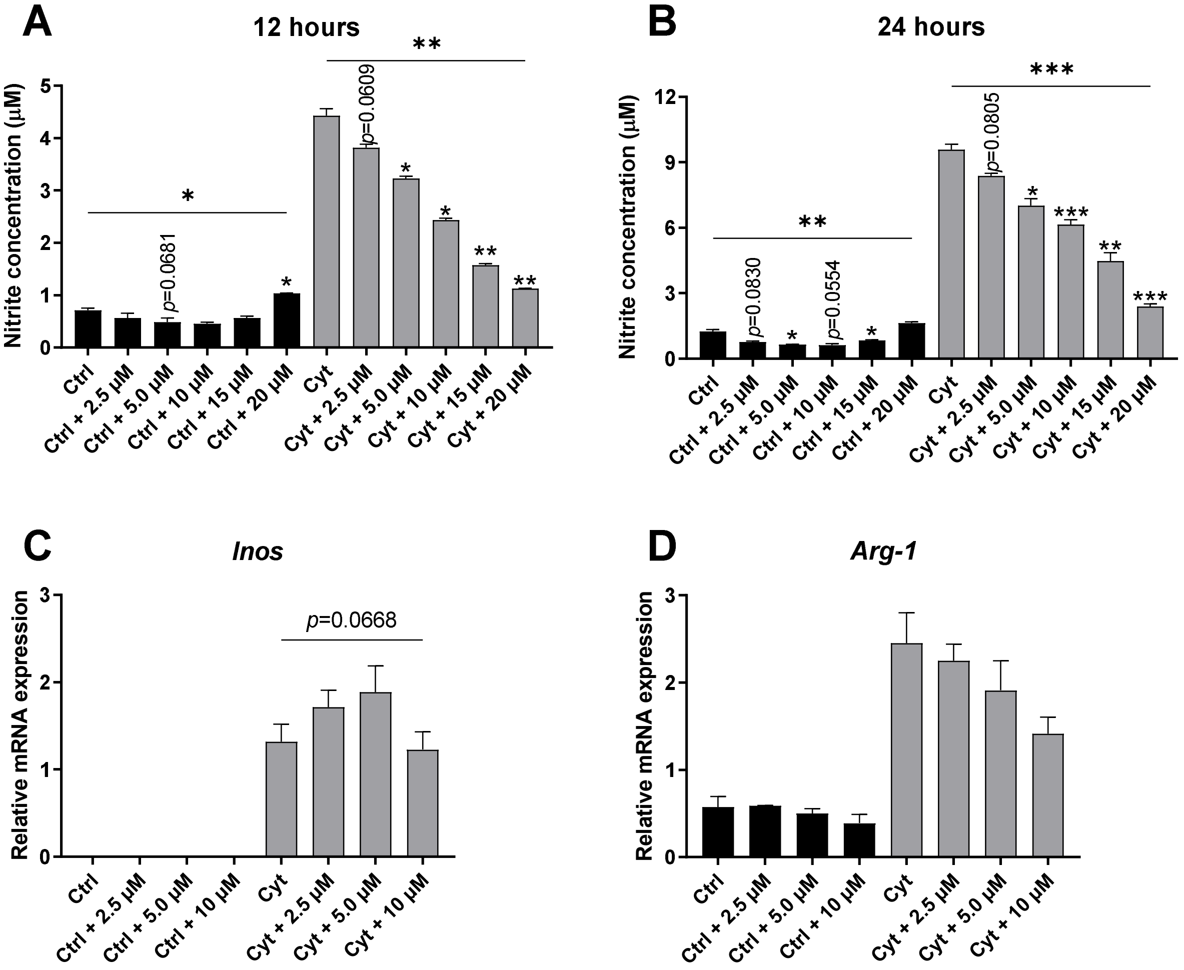

Figure S9. Effects of SR9009 on NO synthesis in non-synchronized INS-1 cells. INS-1 cells were exposed to increasing concentrations of SR9009 alone or in combination with 150 pg/mL mouse IL-1β + 0.1 ng/mL rat IFN-γ (Cyt). Accumulated nitrite was assessed after 12 (**A**) or 24 (**B**) hours exposure. Relative expression of *Inos* (**C**) and *Arg-1* (**D**) were assessed following 12 hours exposure, using *Hprt1* as reference gene. Values are means ± SEM (n = 3). Statistics are one-way ANOVA with p-values represented by symbols above the line and with Dunnett's corrected multiple comparisons to Ctrl (black bars) or to Cyt (grey bars). Significance levels were annotated as follows: *= *p*-value< 0.05, **= *p*-value< 0.01, ***= *p*-value < 0.001.
